# Remote sensing analysis of forest fire impacts on ecosystem productivity, greenhouse gas emissions, and fire risk in Pakistan

**DOI:** 10.1186/s13021-026-00410-y

**Published:** 2026-02-06

**Authors:** Fahad Shahzad, Kaleem Mehmood, Shoaib Ahmad Anees, Muhammad Adnan, Ijlal Haidar, Umarbek Jabbarov, Murodjon Yaxshimuratov, Manuela Oliveira

**Affiliations:** 1 Precision Forestry Key Laboratory of Beijing, Beijing Forestry University, Beijing, 100083 China; 2 Department of Mathematics and Center for Research on Mathematics and its Applications, University of Évora, Évora, Portugal; 3 Key Laboratory for Silviculture and Conservation of Ministry of Education, Beijing Forestry University, Beijing, 100083 P. R. China; 4 Institute of Forest Science, University of Swat, Main Campus Charbagh 19120, Swat, Pakistan; 5 Department of Forestry, The University of Agriculture, Dera Ismail Khan, 29050 Pakistan; 6 State Key Laboratory of Resources and Environmental Information System, Institute of Geographic Sciences and Natural Resources Research, CAS, Beijing, 100101 China; 7 Department of “Accounting and business administration”, Mamun University, Khiva, Uzbekistan; 8 Department of Chemistry, Urganch State University, Urgench, Uzbekistan

**Keywords:** Forest fire, Greenhouse gas emissions, Remote sensing, Burn indices, Machine learning, Ecosystem resilience

## Abstract

This study investigates the spatial variability of forest fire intensity, burn indices, ecosystem productivity, and Greenhouse Gas (GHG) emissions in Pakistan from 2001 to 2023. Using satellite-derived burn indices such as SAVI, LST, NMDI, LSWI, NBR, and MSAVI2, the study examines the relationship between forest fires and net primary productivity (NPP) across diverse ecological regions. The analysis reveals that northern Pakistan, particularly Khyber Pakhtunkhwa and Gilgit-Baltistan, experiences high fire intensity, resulting in significant reductions in NPP and increased emissions of COx, NOx, and CH₄. Central and southern Pakistan, including the arid regions of Balochistan and Sindh, exhibit lower fire intensity but remain vulnerable due to climate-driven dry conditions. The study also applies the ΔNPP/ΔBurn approach to evaluate how changes in burn indices correspond to shifts in NPP, revealing that small increases in fire intensity can lead to substantial ecosystem productivity loss. Additionally, a comparative analysis of Random Forest (RF) and XGBoost machine learning models for fire prediction found RF to be the more accurate model, achieving 88.0% accuracy and a 93.8% AUC score. These findings underscore the importance of developing region-specific fire management strategies to mitigate the ecological and environmental impacts of wildfires. The study highlights the critical need for improved fire prediction, early warning systems, and long-term monitoring of post-fire ecosystem recovery. By drawing comparisons with global research, this study contributes to understanding the broader implications of forest fires on carbon dynamics and ecosystem productivity, providing valuable insights for future fire management policies in Pakistan.

**Supplementary Information:**

The online version contains supplementary material available at 10.1186/s13021-026-00410-y.

## Introduction

NPP and Gross Primary Production (GPP), known as gross carbon fixation from the atmosphere to terrestrial vegetation, and net annual influx of atmospheric CO_2_ into biomass play a key role in the global carbon cycle [55]. These metrics are critical for studying the patterns, dynamics and processes of terrestrial ecosystems [78]. Accurate measurements of GPP and NPP are crucial for addressing diverse ecological issues such as ecosystem modeling applications that depend on fine-scale estimates (respiration partition, photosynthetic efficiency, and land cover class distributions). Historically, GPP and NPP measurements have been based on field observations of specific species at individual sites [27]. While these approaches are detailed, they can be time and labor-intensive and spatially limited due to the natural heterogeneity within ecosystems [21]. Significant advances in remote sensing technology have greatly enhanced our ability to study and assess ecosystems at various scales with higher resolution. Remote sensing has been proven as a cost-effective, efficient and accurate approach for estimating GPP and NPP in regional and or global distributions, over multiple spatial and temporal scales [57, 89]. This novel technological breakthrough is especially useful for studying forest fires (FF) that which are major natural drivers of biodiversity loss [76, 96], depletion of biomass carbon stocks, terrestrial ecosystem productivity reduction within the forests [6], soil fertility degenerations and progressive restriction upon air quality as well as water security accompanied by an increased susceptibility to landslides [14, 43, 69]. FF, as a major disturbance in ecosystems, can have impacts that are both spatially extensive and long-term justifying the need for regional scale understanding of how forest-fires burn intensity is related to ecosystem-level productivity [101].

Over the past several decades, GPP and NPP have been estimated using multiple methods for regional to global scales. These models include: (i) climate productivity models based on the relationship between GPP/NPP and climate factors, such as the Miami model and the Thornthwaite Memorial model [54, 87]; (ii) light use efficiency (LUE) models, like the Carnegie, Ames, Stanford Approach (CASA), which utilize resource balance principles [77], and (iii) eco-physiological process models like CENTURY and the Terrestrial Ecosystem Model (TEM)(McGuire et al., [62, 83]. Each model type has its advantages and limitations: while climate productivity models are simple, they often neglect complex ecological processes and the influence of factors like carbon dioxide, soil moisture, and nutrient conditions [61]. LUE models that use remotely sensed data can be effective for large scales, but they may overlook important ecological dynamics (W.-Q. Zhu et al., [107]. Eco-physiological process models can achieve similar results, but they require intensive computation and need many uncertain input variables [82]. Given these limitations, recent efforts have focused on improving the reliability and feasibility of large-scale ecosystem studies by integrating remote sensing data with eco-physiological process models [84].

Technological advances in remote sensing have likewise revolutionized fire monitoring and assessment, facilitating the identification of ecosystem change [31], as well as providing much improved land use and land cover mapping developments [38]. Satellite imagery plays a crucial role in wildfire management, enabling the mapping of burn severity and informing coordinated fire protection strategies [16, 97]. The dynamics of FF vary across time and space due to interactions among climate, vegetation, topography, and human activities [33, 51]. In Pakistan, the dry sub-tropical climate exacerbates the vulnerability of sub-tropical and temperate forests to fires, with altitudinal variations influencing fire spread [85]. Annually, a significant portion of the country’s forests burn due to natural causes, self-ignition from dry leaves, deforestation, and accidental fires, with over 80% of these fires attributed to anthropogenic activities [35]. Even though there is an adequate explanation of biotic and abiotic aspects responsible for the patterns in fire distribution, Pakistan does not have any actively implemented control strategies at regional and national levels indicative proper ways out [60, 93]. During the last two decades, Machine Learning (ML) models have been used for wildfire prediction and burned area estimation, playing a significant role in fire spread control, damage assessment and the development of predictive models for technological supports [24, 58]. Jain et al. [42] provided a systematic review of major studies on machine-learning applications for fire-related modelling, covering a broad body of literature and summarising key algorithms such as logistic regression, RF, AdaBoost, XGBoost, ANNs, SVMs, and k-NN.

Pakistan has a limited forest resource base (≈ 4.79 million ha; 5.45% of national territory in 2012), and the country is predominantly dry land, with ~ 80% of its land area classified as arid to semi-arid. These baseline conditions increase exposure to prolonged hot–dry periods that favour fuel drying and elevate wildfire susceptibility. *REDD+ - UNFCCC* National hazard assessments further indicate measurable fire-related impacts on woody vegetation; for example, Khyber Pakhtunkhwa has reported the highest tree-cover loss attributable to fires during 2001–2024 (≈ 244 ha yr⁻¹ on average), underscoring that fire constitutes a non-trivial disturbance despite the country’s low overall forest cover [67]. This study aims to address the current research gap by investigating the complex effects of FF on both ecosystem productivity and terrestrial carbon emissions across Pakistan. While previous studies, such as those by [44, 94], have explored similar relationships in regions like India and China, there remains a gap in understanding the specific impacts of FF on ecosystem dynamics and carbon fluxes in Pakistan, especially when considering the interplay between carbon emissions and ecosystem productivity. A key focus of this research is to develop accurate and high-resolution FF incidence mapping to determine the spatially explicit likelihood of FF occurrence. By utilizing open-source, accessible remote sensing datasets, this study provides a methodology that can be widely replicated, ensuring that its findings are relevant in various contexts and adaptable to other regions facing similar challenges. Building on the ΔNPP/Δburn (ΔNPP/ΔIndex) sensitivity framework previously applied in India [86], this study implements and extends the approach for Pakistan (2001–2023) by integrating Landsat-derived fire-scar delineation with FIRMS cross-verification, CASA-based NPP estimation and spatially explicit GHG emission assessment, and RF/XGBoost-based susceptibility modelling with driver interpretation. This integrated framework enables the analysis of the relationship between NPP and burn indices across standard years (2001–2023) and major fire years (2009, 2016, 2021, 2022). The study will explore temporal and spatial variations in NPP and examine the broader consequences of FF on ecosystem productivity and carbon fluxes. Additionally, the research aims to develop predictive models that will improve the accuracy and generalizability of FF risk assessments, providing valuable insights for forest management and climate change mitigation strategies in Pakistan.

## Methodology

### Study area

**Fig. 1:**
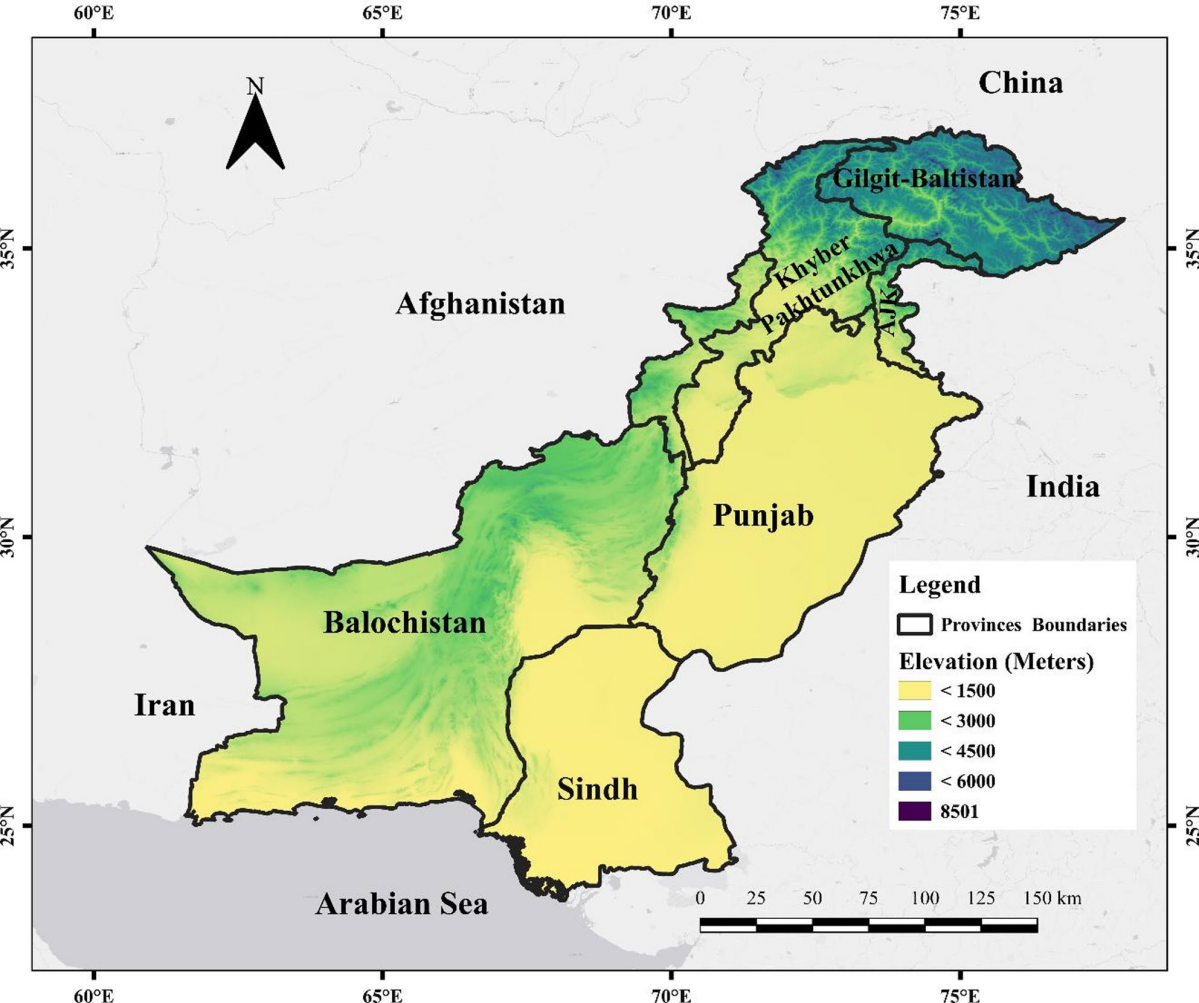
Map of the study area with an overlaid elevation gradient, highlighting the variation in topography across the region

This study focuses on Pakistan from 2001 to 2023. Located in the western part of South Asia, between longitudes 62° to 75°E and latitudes 24° to 37°N, Pakistan spans 875,832 km² with diverse landscapes, from northern mountain ranges to arid southwestern regions (Fig. 1) [79]. The study domain spans a strong elevation and physiographic gradient, including the Hindu Kush–Karakoram–Himalaya ranges in the north, the Indus alluvial plains, the Potohar Plateau and salt range belt, the Balochistan Plateau, and the Thar/Cholistan desert systems. Despite its geographic diversity, the country’s forest cover is only 4.5% [64], with 223,800 fire incidents recorded, 30,394 of which occurred in forested areas (MCD12Q1 MODIS LC_type 5 data). Pakistan’s economy heavily relies on agriculture, and it is situated within the South Asian Ecological Zone [39, 73]. The country experiences four seasons: dry winter, hot spring, monsoon, and post-monsoon [89]. Climatic conditions vary markedly across the country, with high precipitation in the northern foothills and mountain zones and very low rainfall in the arid south-west; monsoon rainfall dominates the hydrological regime in much of the east and north, while winter precipitation associated with western disturbances is more influential in the west and north-west. These coupled gradients in climate, fuels, and land use provide a strong basis for spatially explicit assessment of fire susceptibility and post-fire ecosystem response using satellite observations [46]. Remote sensing advancements offer opportunities to improve fire prevention measures and wildfire prediction models[7] .

### Data sources

This study integrated multi-source spatial and temporal datasets for evaluating forest fire intensity, vegetation dynamics, and GHG emissions across Pakistan from 2001 to 2023. The data sources comprised Landsat satellite imagery for vegetation indices and Land Surface Temperature (LST), fire occurrence data from FIRMS, climate variables from TerraClimate, auxiliary datasets for topographic, and socioeconomic characterization. Landsat surface reflectance products (Collection 2 Level 2) from Landsat 5, 7, and 8 satellites were used to derive vegetation indices sensitive to fire disturbance, including the Normalized Burn Ratio (NBR) [30], Soil Adjusted Vegetation Index (SAVI) [72], Land Surface Water Index (LSWI) [59], Normalized Multi-Band Drought Index (NMDI) [68], and Modified SAVI2 (MSAVI2) [5]. Land Surface Temperature (LST) was obtained in Google Earth Engine from the Landsat Collection 2 Level-2 Surface Temperature (ST) product and processed using a radiative transfer–based approach with atmospheric correction and surface emissivity parameterisation; full implementation details are provided in Supplementary Sect. "Methodology" (Ermida et al., 2020; Soomro et al., [95]. The Landsat archive provides a spatial resolution of 30 m with a revisit time of 16 days, enabling high-resolution monitoring of vegetation dynamics and post-fire conditions [8]. The derivation of Vegetation Indices and LST are briefly explained in supplementary material Sect. "Methodology". Land-cover stratification and masking were implemented using the MODIS Land Cover Type product (MCD12Q1; LC_Type5; 500 m, annual), which was used to (i) restrict fire analyses to forest/vegetated fuel classes and (ii) exclude non-burnable classes (e.g., urban/built-up and water) during susceptibility mapping.

Active fire occurrence data were acquired from NASA’s Fire Information for Resource Management System (FIRMS), which provides detailed spatiotemporal information on fire locations, detection time, and associated confidence levels [20]. These active fire points served as a reference dataset to validate the fire events and burned area extent derived from the Landsat-based NBR analysis [49]. Specifically, annual fire counts extracted from the NBR-based burn indices were cross-verified against the FIRMS active fire records to ensure the accuracy and consistency of fire occurrence detection across the study period. For the vegetation category, Landsat-based vegetation indices such as the Enhanced Vegetation Index (EVI) and the Normalized Difference Vegetation Index (NDVI) were employed to assess the quantity and quality of vegetation cover. These indices offer insights into vegetation health, state, and moisture content through spectral observations [65]. In addition to EVI and NDVI, Leaf Area Index (LAI) was obtained from the MODIS LAI/FPAR 8-day composite product (MCD15A2H/MOD15A2H, Collection 6.1; 500 m) via Google Earth Engine. Annual LAI composites were generated (median) for 2001–2023 and harmonised to the analysis grid prior to modelling [28]. Together, these Landsat-based indices provide a comprehensive understanding of vegetation dynamics across diverse landscapes. Climatic variables, including precipitation, temperature, vapor pressure, and wind speed, were acquired from the TerraClimate dataset with a spatial resolution of ~ 4 km and monthly temporal coverage [88]. These variables supported NPP estimation and the assessment of environmental stress on ecosystems. Topographic attributes such as elevation, slope, and aspect were derived from the Shuttle Radar Topography Mission (SRTM) Digital Elevation Model (DEM) at 30-meter resolution [32]. Population density data were obtained from the WorldPop database to account for anthropogenic pressures influencing fire occurrences [22]. All datasets were reprojected to a uniform coordinate system (WGS84) and resampled to a common spatial resolution of 30 m (continuous variables: bilinear; categorical land-cover classes: nearest-neighbour) to ensure consistency across analyses. Atmospheric pollution data, including sulfur dioxide (SO₂) and nitrogen dioxide (NO₂) concentrations, were obtained from the Sentinel-5P Near Real-Time (NRTI) dataset via Google Earth Engine (GEE). As Sentinel-5P is available from 2018 onwards, these variables were analysed for the 2018–2023 period to relate recent fire hotspots with air-quality patterns [86]. The integration of multi-source datasets provided a comprehensive spatiotemporal framework to evaluate the linkages between forest fire dynamics, vegetation productivity, and carbon emissions across Pakistan’s diverse ecological regions All data sources used in this study are detailed in Supplementary Table S1.

### Estimation of fire counts from NBR and validation with FIRMS data

Annual NBR composites were generated from pre- and post-fire Landsat images. The difference between NBR (dNBR) was calculated by subtracting the post-fire NBR from the pre-fire NBR to capture vegetation loss due to burning [41]. A conservative burned-area threshold of dNBR > 0.1 was applied, consistent with common dNBR conventions for separating unburned/low-change pixels from burned conditions and refined based on regional spectral response and visual inspection in GEE [34]. Burned patches were extracted using connected component analysis, and annual “fire counts” were quantified as the number of spatially discrete burned patches (fire scars) per year rather than independent ignition events [74]. Validation used NASA FIRMS active-fire detections by matching FIRMS points with Landsat-derived burned patches within the same fire year using a 500 m proximity buffer; multiple FIRMS points within a single Landsat patch were treated as a single match. A summary of match/omission/commission rates is reported in the main text, with full validation details provided in Supplementary Sect. 2. In this study, “fire counts” represent the annual number of spatially discrete burned patches (fire scars) derived from Landsat, rather than the number of independent ignition events. Importantly, fire counts were extracted specifically from forested areas identified using the MODIS Land Cover Type Product (MCD12Q1), LC_type 5, which classifies the landscape into 12 distinct land cover types [38]. From these, six classes were selected as forest land for fire count extraction: Evergreen Needleleaf Trees (ENF), Evergreen Broadleaf Trees (EBF), Deciduous Needleleaf Trees (DNT), Deciduous Broadleaf Trees (DBT), Shrubland, and Grassland, all mapped at a 500-meter resolution. This stratification ensured that fire counts represented only forest-related ecosystems, excluding non-forest land covers. The accuracy of Landsat-derived fire counts was validated using the active fire dataset from FIRMS, which provides fire detection points based on satellite-derived thermal anomalies [36]. A spatial overlay analysis was conducted to match NBR-derived fire clusters with FIRMS fire points within the corresponding fire year. The validation results are available in supplementary material Sect. 2. To address the spatial resolution difference between Landsat (30 m) and FIRMS (1 km), a proximity buffer of 500 m was applied around FIRMS fire points during the validation process. FIRMS detections were used only to validate the Landsat-derived burned patches; multiple FIRMS points falling within the same Landsat burned patch were treated as a single match and did not increase the patch-based fire count. We note that a large contiguous burned patch may contain multiple ignitions that cannot be separated reliably using annual Landsat composites; therefore, the metric should be interpreted as burned-patch (fire-scar) frequency rather than event frequency. Annual fire activity was quantified as the number of Landsat-derived burned patches (fire scars) within the vegetated-fuel mask for each year (2001–2023). Years with annual fire counts exceeding 2000 patches were classified as “fire years”, while the remaining years were designated as “non-fire years” for subsequent analysis. Using this criterion, the identified fire years were 2009, 2016, 2021, and 2022, and all other years (2001–2023) were treated as non-fire years. The validation confirmed a strong spatial correspondence between the NBR-derived fire counts and FIRMS fire detections, supporting the reliability of the fire count estimation approach employed in this study.

### Quantification of NPP, carbon emissions, and greenhouse gas emissions

The study aims to estimate NPP in Pakistan using the Carnegie–Ames–Stanford Approach (CASA) model, focusing on six forest land classes selected for fire count extraction: ENF, EBF, DNT, DBT, Shrubland, and Grassland. Remote sensing data, including the NDVI, solar radiation, and temperature, are utilized in this approach [77]. NDVI is used to estimate the FPAR absorbed by the vegetation, and solar radiation data is used to calculate Absorbed Photosynthetically Active Radiation (APAR). Additionally, temperature data is incorporated for environmental limitations on light energy utilization efficiency, which is a crucial factor in the model [105]. The APAR for each pixel at a given time is calculated using the Eq. (1):

1 $$\:\:{\mathrm{A}\mathrm{P}\mathrm{A}\mathrm{R}}_{\left(x,\:t\right)}=0.5\:\times\:\:{Solar\:Radiation}_{\left(x,\:t\right)}\times\:\:{\mathrm{F}\mathrm{P}\mathrm{A}\mathrm{R}}_{\left(x,\:t\right)}\:\:\:\:\:\:\:\:\:\:\:\:\:\:\:\:\:\:\:\:\:\:\:$$

where, constant $\:0.5\:$ represents the fraction of solar radiation that can be used by vegetation as effective solar radiation. $\:{Solar\:Radiation}_{(x,\:t)}$ is the total solar radiation for pixel $\:x$ at time $\:t$, and $\:{\mathrm{F}\mathrm{P}\mathrm{A}\mathrm{R}}_{(x,\:t)}\:$ is the fraction of vegetation APAR. The $\:{\mathrm{F}\mathrm{P}\mathrm{A}\mathrm{R}}_{(x,\:t)}\:$is calculated based on the NDVI data, as FPAR has a linear relationship with NDVI. The relationship between FPAR and NDVI can be estimated according to the Equ: 2.

2 $$\:{\mathrm{F}\mathrm{P}\mathrm{A}\mathrm{R}}_{\left(x,\:t\right)}\:=\:\mathrm{a}\:\times\:\:{NDVI}_{\left(x,\:t\right)}+\mathrm{b}\:\:\:\:\:\:\:\:\:\:\:\:\:\:\:\:\:\:\:\:\:\:\:\:\:\:\:\:\:\:\:\:\:\:$$

where we set $\:a=1.164$ and $\:b=-0.143$ following the empirically supported linear relationship between NDVI and FAPAR reported by Myneni and Williams [70].

FPAR values were subsequently constrained to the commonly used physical bounds [0.001, 0.95] to prevent unrealistic values in very low- or high-NDVI conditions. The light use efficiency (**ε**) is calculated as:

3 $$\:{{\varepsilon\:}}_{(x,\:t)}\:=\:{{\varepsilon\:}}_{max}\:\times\:\:{T{\varepsilon\:}}_{1(x,t)}\:\times\:\:{T{\varepsilon\:}}_{2(x,t)}\:\times\:\:{W{\varepsilon\:}}_{\left(x,t\right)}\:\:\:\:\:\:\:\:\:\:\:\:\:\:\:\:\:$$

where, $\:{T{\varepsilon}}_{1(x,t)}$ accounts for the influence of extreme temperatures, both high and low, on vegetation, while $\:{T{\varepsilon}}_{2(x,t)}\:$ reflects the ambient temperature that the vegetation experiences. The term $\:{W{\varepsilon}}_{\left(x,t\right)\:}$describes how humidity conditions impact the efficiency of light energy utilization by plants. $\:{{\varepsilon}}_{max}$ represents the maximum efficiency at which vegetation can convert light energy into plant biomass under optimal conditions. This parameter is critical for calculating light use efficiency in the CASA model and varies according to the specific vegetation type and regional environmental factors [37, 103]. In this study, we have used the $\:{\varepsilon}_{max}$values parameterised by vegetation functional type and adopted from established CASA applications [106]. Accordingly, $\:{\varepsilon}_{max}$was assigned by land-cover class was assigned as follows: 0.375 for ENF, 0.480 for EBF, 0.480 for DNT, 0.682 for DBT, 0.433 for Shrubland, and 0.536 for Grassland. These class**-**specific values were selected to maintain consistency with prior CASA calibrations and to reflect known differences in maximum light-use efficiency among vegetation types [106].

Once $\:{\mathrm{A}\mathrm{P}\mathrm{A}\mathrm{R}}_{(x,\:t)}$ and $\:{{\varepsilon\:}}_{(x,\:t)}$are determined, the NPP is calculated by multiplying the two values:

4 $$\:{\mathrm{N}\mathrm{P}\mathrm{P}}_{(x,\:t)}=\:{{\varepsilon\:}}_{(x,\:t)}\times\:\:{\mathrm{A}\mathrm{P}\mathrm{A}\mathrm{R}}_{(x,\:t)}\:\:\:\:\:\:\:\:\:\:\:\:\:\:\:\:\:\:\:\:\:\:\:\:\:\:\:\:\:$$

This equation calculates the NPP for each pixel over time, across six vegetation classes ENF, EBF, DNT, DBT, Shrub, and Grassland. NPP is computed separately for each vegetation type, providing detailed estimates for each class across Pakistan. The resulting NPP values are then aggregated to generate a regional estimate, offering valuable insights into vegetation productivity and biomass accumulation across Pakistan.

The quantification of GHG emissions in this study involved estimating the spatiotemporal emissions of key GHG (COx, CH_4_, N_2_O, and particulate matter) using the NPP approach. NPP was used to evaluate the environmental effect of FF, particularly focusing on the loss of natural resources in substantially enriched ecosystems. To calculate the amount of carbon released due to FF, the study followed the IPCC procedures for national GHG records. The formula used was [23]; Intergovernmental Panel on Climate Change (IPCC) [40]), :

5 $$\:C=\:{\Delta\:}\mathrm{B}\mathrm{i}\mathrm{o}\mathrm{m}\mathrm{a}\mathrm{s}\mathrm{s}\:\times\:\:0.9\:\times\:\:0.45\:$$

In this formula, $\:C$ (in grams of carbon) represents the amount of carbon released, $\:{\Delta\:}\mathrm{B}\mathrm{i}\mathrm{o}\mathrm{m}\mathrm{a}\mathrm{s}\mathrm{s}$ refers the difference in biomass observed between normal years and fire years, 0.9 is the fraction of biomass that is oxidized on site during a fire, and 0.45 represents the carbon contented fraction in the biomass [66].

For calculating the emissions of specific carbon compounds such as CO_2_, CH_4_, and CO, the following formula was applied:

6 $$\:{E}_{j}=\:{\varepsilon\:}_{j}\times\:\:{\delta\:}_{j}\times\:\mathrm{C}\:$$

In this equation, $\:{E}_{j}$ represents the emission of compounds such as CO_2_, CH_4_, and CO, $\:{\varepsilon\:}_{j}$ represents the proportion of the total carbon released in the form of a specific compound, and $\:{\delta\:}_{j}$ is the conversion factor that translates carbon emissions into the specific compound. For CO_2_, CH_4_, and CO, the values used were ϵj = 0.888 for CO_2_, 0.012 for CH_4_, and 0.1 for CO, while the conversion factors δj​ were 3.67 for CO_2_, 1.33 for CH_4_, and 2.33 for CO [23]; Sannigrahi et al., [86, 104]. The validation of CASA-Derived NPP against MODIS NPP are detailed in supplementary material Sect. 2.

Similarly, the emissions of nitrogen compounds, such as NO_2_ and NOx, were calculated using:

7 $$\:N=\:{\gamma\:}^{*}C$$

and

8 $$\:{E}_{j}=\:{\varepsilon\:}_{j}\times\:\:{\delta\:}_{j}\times\:\mathrm{N}$$

Where, $\:N$ is the amount of nitrogen emitted, and $\:{\gamma\:}^{*}$ is the ratio of emitted nitrogen to carbon. The specific emissions of NO_2_ and NOx were then calculated using the values $\:{\epsilon\:}_{j}$​= 0.007 for NO_2_ and 0.012 for NOx, and $\:{\delta\:}_{j}$conversion factors of 1.57 for NO_2_ and 2.14 for NOx. These mathematical formulas and values enabled a detailed and accurate estimation of the GHG emissions resulting from FF, allowing for a better understanding of their environmental impact (Intergovernmental Panel on Climate Change (IPCC) [40],; Meinshausen et al., [66, 104].

### Spatial analysis of fire Intensity, vegetation response, and atmospheric pollution

The spatial patterns of Forest Fire Intensity (FFI) were quantified using Kernel Density Estimation (KDE), implemented through the kernel density and point density tools available in QGIS 3.42 [50]. KDE is a widely applied non-parametric technique that estimates the spatial probability density of point-based events over a continuous surface and is particularly suitable for representing fire occurrence concentrations across heterogeneous landscapes [48]. The general mathematical formulation of KDE is expressed as:

9 $$\:\widehat{f}\left(x\right)=\:\frac{1}{{nh}^{2}}\:{\sum}_{i=1}^{n}K\left(\frac{x-\:{x}_{i}}{h}\right)$$

where $\:\widehat{f}\left(x\right)$ is the estimated density at location $\:x$, $\:n$ is the total number of fire event points, $\:h$ corresponds to bandwidth (search radius), $\:K$ is the kernel function (Gaussian kernel was applied), and xi​ is the location of each fire event point. A search radius (bandwidth) of 500 m was applied in the KDE analysis, corresponding to the spatial characteristics of the Landsat-derived burned area dataset (30 m resolution). This search radius was selected to capture the localized concentration of fire activity while minimizing the risk of over-smoothing or merging independent fire events. The choice of 500 m is consistent with previous studies that applied KDE for forest fire intensity mapping in heterogeneous landscapes [50]; Liu et al., [56]. This KDE approach generated continuous surface maps of FFI, highlighting fire-prone hotspots and spatial gradients of fire occurrence intensity [52]. In addition, a two-dimensional kernel density analysis (2D-KDE) was conducted in R version 4.3.0 to examine the density-based relationship between NPP and fire-sensitive burn indices (LST, NMDI, LSWI, SAVI, NBR, and MSAVI2). This method enabled the visualization of joint density distributions between NPP and burn indices, facilitating the identification of regions with high vegetation sensitivity to fire disturbances during fire and non-fire years [52, 53]. Furthermore, Sentinel-5P-derived SO₂ and NO₂ concentration layers were processed within the GEE environment and spatially overlaid with fire intensity zones and vegetation productivity maps [86]. This analysis aimed to explore potential linkages between atmospheric pollution levels and fire-impacted ecosystems across the study area.

### Random forest

RF and XGBoost were selected because tree-based ensemble learners are well suited to wildfire susceptibility modelling with heterogeneous and non-linear predictors (topography, climate/fire-weather, vegetation indices, and human-pressure proxies). Both algorithms have demonstrated strong predictive performance and operational utility in susceptibility/risk mapping studies, and they are increasingly used in wildfire probability applications, including Pakistan-focused analyses [2–4, 75].The RF and XGBoost models were trained using *n* = 12 explanatory variables representing vegetation condition, climate/fire-weather, topography, and human pressure. The features included elevation, slope, aspect (SRTM-derived); precipitation, temperature, vapour pressure, and wind speed (TerraClimate); NDVI, EVI, LAI, and LST (satellite-derived); and population density (WorldPop) (details in Supplementary Table S1). LAI was included as an explanatory predictor to represent vegetation density (fuel availability) in the fire susceptibility models. The RF model, an ensemble method based on decision trees, enhances prediction accuracy and robustness by aggregating multiple decision trees [18]. In this study, RF is used for classification tasks, with hyperparameter tuning integrated to optimize its performance. Key hyperparameters such as the number of trees (ntree), maximum depth (max_depth), and the number of features considered for splitting (max_features) were tuned. A grid-search was implemented over predefined ranges (ntree: 300–1500; max_depth: 10–60; max_features: {√p, p/3, p/2}), and model selection was based on minimising out-of-bag (OOB) error, which provides an internal, unbiased generalisation estimate for bootstrap-aggregated trees [25]. To further stabilise performance estimates, the optimal configuration was confirmed using k-fold cross-validation (k = 5) on the training set, and final metrics were reported on the held-out test set. This approach ensures the model balances computational cost and accuracy, preventing overfitting or underfitting [91]. This methodology allows for building an RF model that is both accurate and computationally efficient, while providing insights into the relevance of various input variables [9, 17]. Further, variable importance was assessed using SHAP values, which provide an interpretable view of feature contributions by quantifying the impact of each variable on individual predictions [65].

### Extreme gradient boost

XGBoost, introduced by [15], is a Gradient-Boosting Decision Tree framework designed for computational efficiency and generalisation. In this study, hyperparameter tuning was performed to enhance the performance of the XGBoost model. The key hyperparameters included the number of trees (n_estimators), the learning rate (eta), and the maximum depth of each tree (max_depth). A grid-search with 5-fold cross-validation was applied to tune n_estimators (200–800), eta (0.05–0.30), and max_depth (3–12), with additional regularisation terms (subsample and colsample_bytree) tuned within 0.6–1.0 to reduce overfitting. The final hyperparameter set was selected based on the best mean cross-validated AUC, and final model performance was evaluated on the held-out test set using accuracy, precision, recall, F1-score and ROC–AUC [10, 63].

## Results

### Relationships between forest fire intensity, burn indices, and ecosystem productivity

**Fig. 2:**
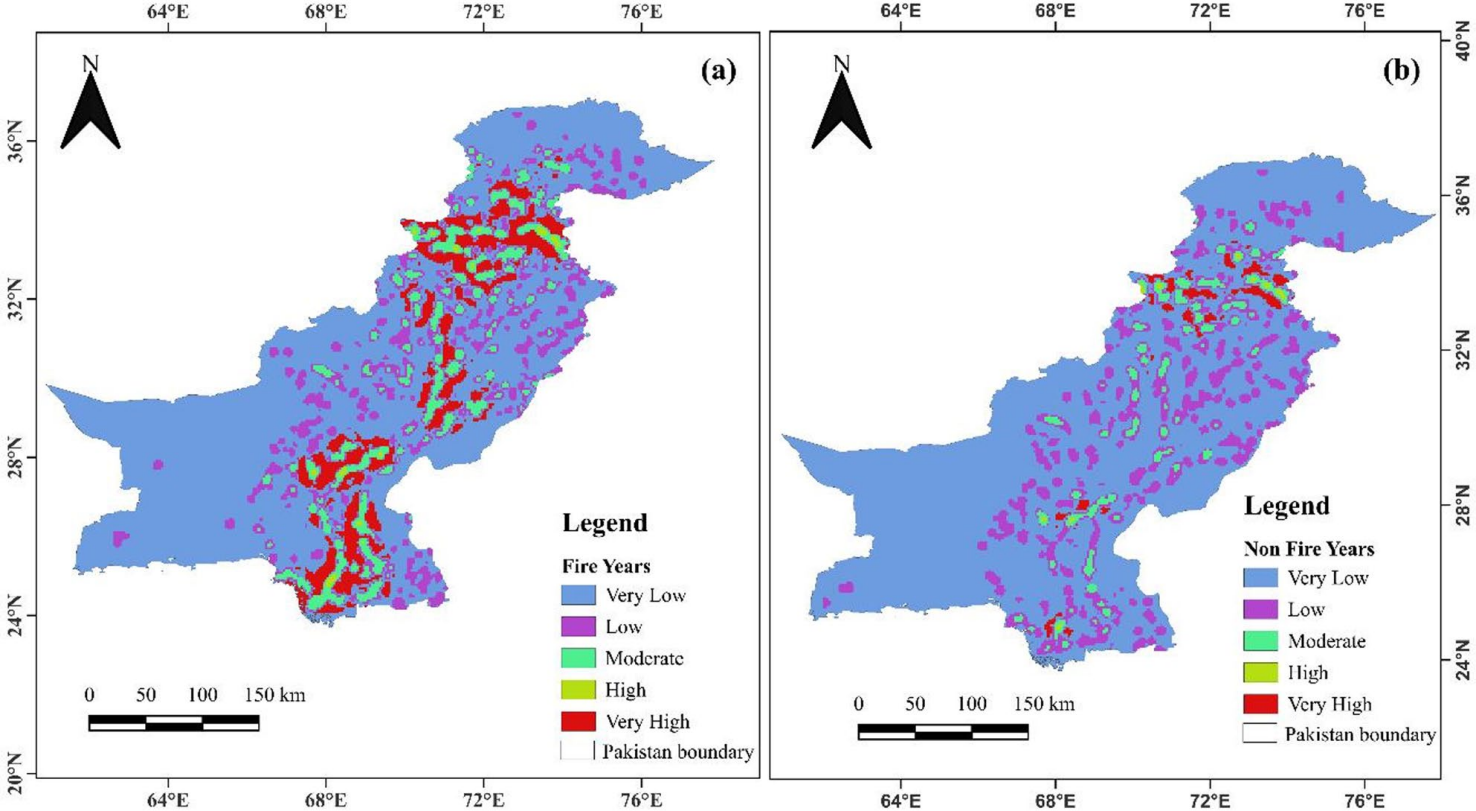
FFI calculated by point and kernel density for (**a**) fire years and (**b**) non fire years

The spatial distribution of Fire Frequency Intensity (FFI) in both fire and non-fire years (Fig. 2) shows that higher observed fire intensity is common in northern regions like Khyber Pakhtunkhwa (KPK) and Gilgit-Baltistan (GB), where continuous forest fuels and complex mountainous terrain can amplify fire spread and increase impacts when burning occurs. In fire years, very high intensity zones cover 0.43%, high intensity zones 3.4%, moderate intensity zones 16.86%, and low intensity zones dominate at 77.5%. In non-fire years, very high intensity zones drop to 0.26%, high intensity zones increase to 9.41%, and low intensity zones cover 66.81%, with 10.33% of the area classified as very low intensity (Table S2). In central regions like Punjab and parts of KPK, and southern areas such as Balochistan and Sindh, fire susceptibility can be elevated under hot–dry and wind-favourable conditions, particularly across shrublands/grasslands and degraded rangelands; therefore, high susceptibility in the south does not necessarily imply dense forest cover, but rather indicates favourable ignition and spread conditions in vegetated fuel landscapes. The variation in fire intensity is shown by standard deviation results (Figure S1), with lower values (0–13,804.09) indicating consistent fire behavior, while higher values (up to 259,115) reflect more erratic fire activity. This variability emphasizes the need for region-specific fire management strategies.

**Fig. 3:**
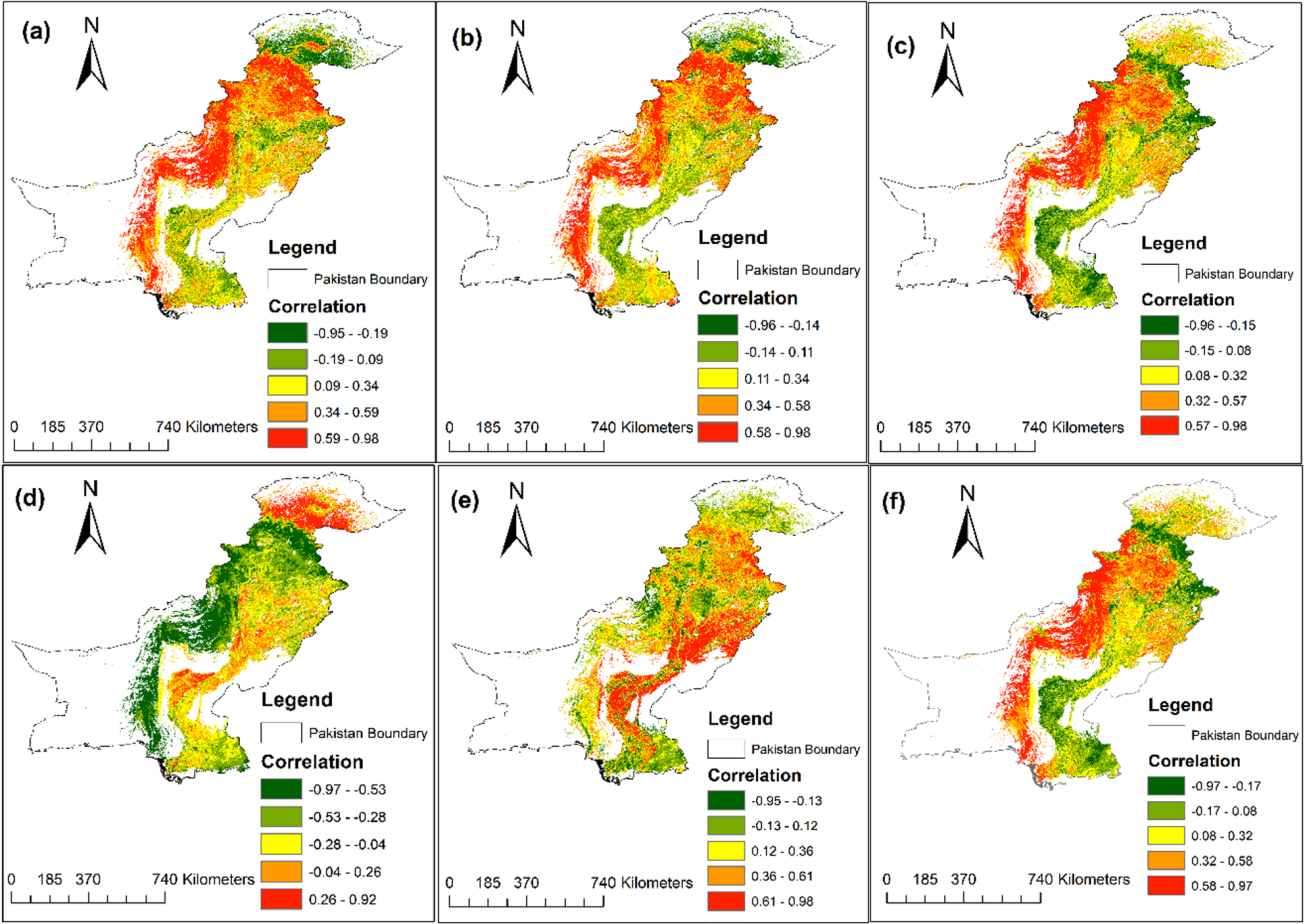
Spatial correlation between NPP and burn indices: (**a**) NBR, (**b**) LSWI, (**c**) SAVI, (**d**) LST, (**e**) NDMI, and (**f**) MSAVI2 with NPP

The association between NPP and burn indices was analyzed to explore the link between ecosystem productivity and fire intensity (Fig. 3). Strong negative correlations were found in forested zones, particularly in northern Pakistan (GB, KPK, AJK), indicating high vulnerability to wildfires. In these areas, dense forests and mountainous terrain lead to a significant decrease in NPP due to frequent burn events. Maps in Fig. 3 show extensive areas of negative NPP–index correlation in the northern forested belt, particularly for LSWI, NBR, and MSAVI2, indicating that stronger burn/moisture stress signals are associated with reduced ecosystem productivity. In central regions like Punjab and parts of KPK, the negative correlation is weaker, suggesting more resilience to fire impacts. Southern regions, including Sindh and Balochistan, show varying patterns, with some coastal and arid areas showing localised positive correlations that may reflect rapid post-fire regrowth/resprouting in shrublands, short-term greening after low severity burning, and/or seasonal vegetation dynamics in managed and irrigated landscapes. These positive correlations should therefore be interpreted cautiously as correlation-based responses rather than direct evidence of beneficial fire effects. However, forested areas in southern Balochistan still display negative correlations, indicating ongoing vulnerability. Western Pakistan, with its sparse vegetation, shows inconsistent correlation patterns, while eastern agricultural zones display moderate negative correlations due to controlled post-harvest burning.

At the national pooled level, the correlation matrix (Fig. S2) indicates that NPP is most strongly associated with SAVI (*r* = 0.28, *p* < 0.05) and MSAVI2 (*r* = 0.27, *p* < 0.05), followed by LSWI and NMDI (*r* = 0.25 for both), whereas NBR shows a comparatively weaker association (*r* = 0.21). LST exhibits a negative association with NPP (*r* = − 0.35), consistent with thermal stress conditions. Notably, several indices are highly inter-correlated (e.g., LSWI–NBR *r* ≈ 0.97), suggesting partial redundancy; therefore, interpretation emphasises the most diagnostically informative indices (NBR/LSWI for burn/moisture constraints and SAVI/MSAVI2 for soil-adjusted vegetation response).The northern belt is characterised by higher observed fire intensity and stronger negative NPP responses in dense forests, whereas parts of the south show higher modelled susceptibility under dry fire-weather conditions across non-forest vegetated fuels, with more heterogeneous NPP–index relationships. The Statistical validity of the parameter evaluations at the 0.05 significance level highlights the connection between FF-induced biodiversity loss and the associated trade-offs on key controlling and supporting ecosystem services, such as carbon sequestration and GHG regulation, which are assessed in this study (Figure S2).

### Assessing fire impact on vegetation productivity using the ΔIndex/ΔNPP approach across Pakistan (2001–2023)

**Fig. 4:**
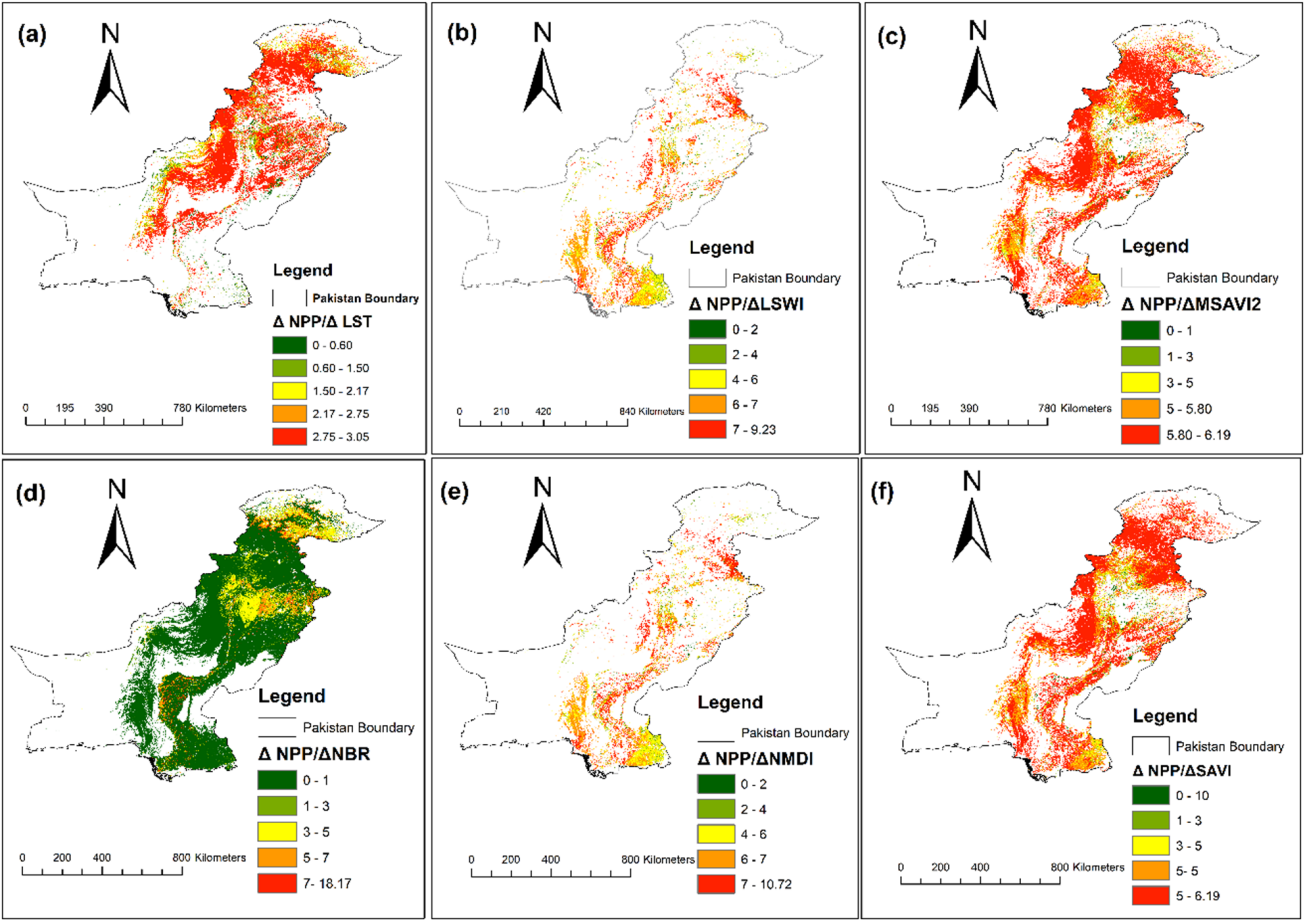
Variation in NPP relative to Shifts in burn indices: (**a**) ΔNPP/ΔLST, (**b**) ΔNPP/ΔLSWI, (**c**) ΔNPP/ΔMSAVI2, (**d**) ΔNPP/ΔNBR, (**e**) ΔNPP/ΔNDMI, and (**f**) ΔNPP/ΔSAVI

The spatial relationship between NPP and burn indices was analyzed using the ΔIndex/ΔNPP approach (Fig. 4), which explores how changes in burn indices correspond to shifts in NPP. This method offers a deeper understanding of how fire dynamics affect ecosystem productivity, identifying regions where NPP is more sensitive to fire events. Higher ΔNPP/ΔIndex values, which represent stronger spatial linkages between burn indices and NPP, are primarily concentrated in areas with higher fire frequency and FFI. This reinforces the utility of satellite-derived burn indices in spatially identifying fire-vulnerable zones and their impact on vegetation productivity.

In northern Pakistan, particularly the forested regions of GB, KPK, and Azad AJK, we observe consistently high ΔIndex/ΔNPP values. This reflects the noticeable sensitivity of NPP to fire disturbances in these dense forests, where fire events whether wildfires or controlled burns lead to substantial reductions in productivity. These elevated values suggest that even small variations in burn indices have significant impact on NPP, underscoring the vulnerability of these ecosystems to fire-related disturbances. In central regions of Pakistan, including Punjab and parts of KPK, the relationship is more moderate, as shown by lower ΔIndex/ΔNPP values. These regions comprise a mix of agricultural land and forested land, where fire management practices such as controlled burns are more prevalent. Consequently, the impact of fire on NPP is less severe, though some zones near forest still exhibit higher sensitivity, necessitating continued attention to fire management in these areas. In southern regions of Pakistan, particularly in Sindh and Balochistan, the ΔIndex/ΔNPP values display more variability. Coastal and arid regions tend to have lower values, suggesting a weaker relationship between fire disturbances and NPP, likely due to the prevalence of fire tolerant shrubland and sparse vegetation. However, in parts of southern Balochistan, isolated patches with higher ΔIndex/ΔNPP values indicate areas of increased vulnerability, where fire events can still significantly reduce productivity, especially in denser vegetation zones.

The arid western zones of Balochistan consistently show low ΔIndex/ΔNPP values, reflecting the minimal impact of fire disturbances on the already sparse vegetation in these areas. Similarly, in eastern Pakistan, dominated by agricultural land, the response of NPP to fire is muted; controlled post-harvest fires do not significantly affect productivity. This reflects the ability of these ecosystems’ capacity to withstand or quickly recover from fire events. In summary, the ΔIndex/ΔNPP analysis provides valuable insights into how fire dynamics influence vegetation productivity across Pakistan. High values in northern regions reveal the critical impact of fire on forested ecosystems, while lower values in central and southern regions reflect varying levels of resilience to fire disturbances. These findings highlight the importance of targeted fire management strategies, especially in areas where high ΔIndex/ΔNPP values indicate sharp sensitivity to fire.

### Carbon sequestration and greenhouse gases emissions

**Fig. 5:**
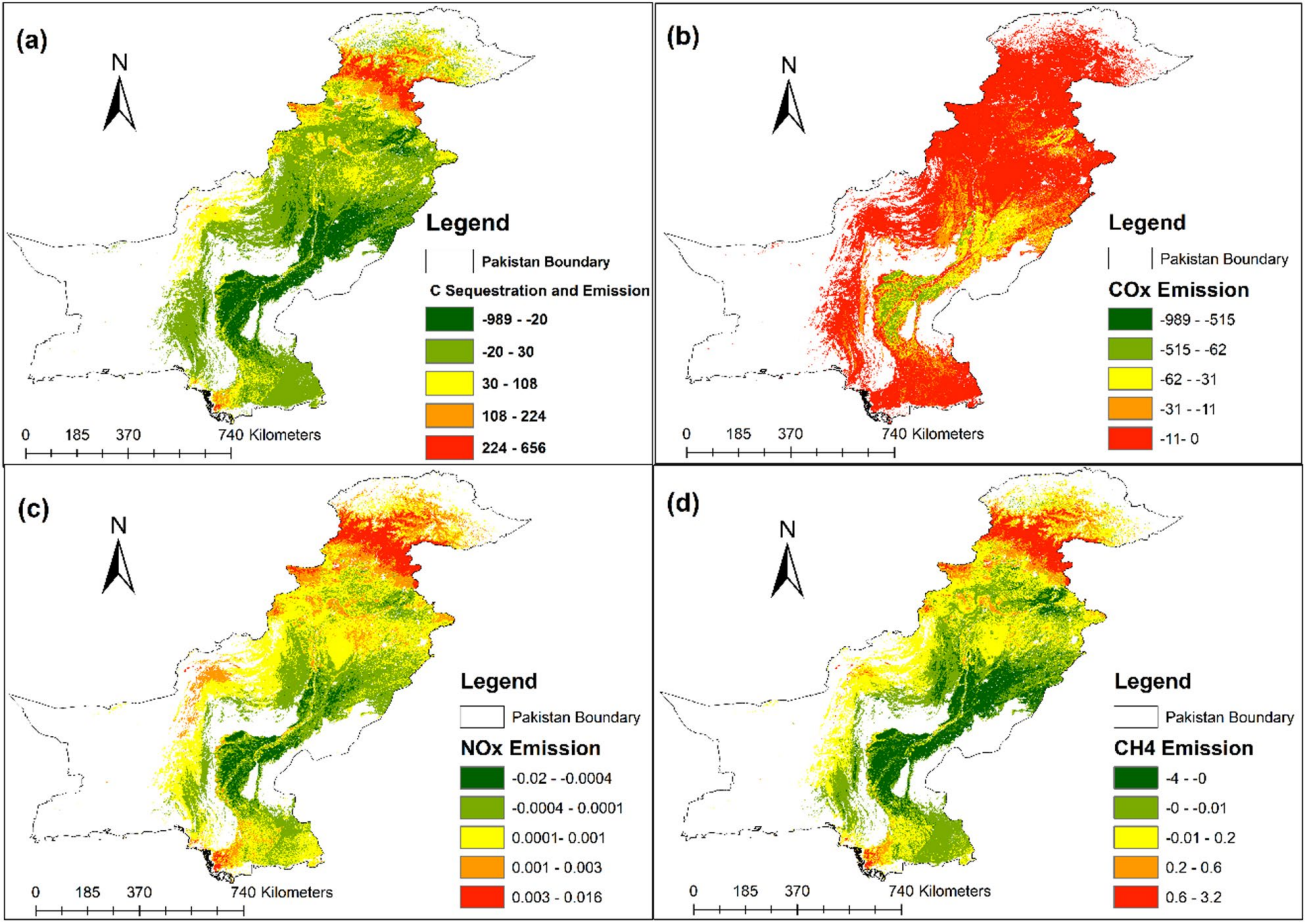
Fire-attributable carbon balance and biomass-burning emissions across Pakistan derived using the CASA–IPCC framework (Eqs. 5–6): (**a**) carbon sequestration and fire-related carbon release, (**b**) COx/CO₂ emissions, (**c**) NOx emissions and (**d**) CH₄ emissions. Emissions are estimated from $\:{\Delta\:}$Biomass between non-fire (baseline) and fire-year conditions; therefore, the maps represent fire-associated changes rather than continuous urban/industrial emissions

The analysis of FF impacts on carbon sequestration and emissions across Pakistan, as shown in Fig. 5, highlights substantial regional variability. Carbon sequestration and emissions were modeled using the CASA model (Figure S3). It is important to note that the emissions mapped in Fig. 5 represent fire-attributable biomass-burning emissions, not continuous fossil-fuel emissions from urban or industrial sources. Following the IPCC-based approach (Eqs. 5–6), carbon release was estimated from $\:{\Delta\:}$Biomass derived as the difference between non-fire (baseline) and fire-year conditions and then converted to individual gas species (COx/CO₂, NOx, CH₄). Therefore, the mapped patterns reflect fire-driven emission pulses associated with biomass loss where burning occurred. The spatial distribution of carbon sequestration and fire-attributable emission patterns shows that regions in northern Pakistan exhibit high sequestration potential, particularly in forested areas. The sequestration values ranged from − 30 to 389 gC/m². In contrast, high emission zones are concentrated in the southern and central parts of the country, with values reaching up to 224–666 gC/m², indicating substantial carbon release attributable to FF (Fig. 5a). The CO₂/COx emissions indicate fire-attributable increases across several regions, with the red and orange zones (11–515 gCO₂ m⁻²) highlighting areas most affected by FF and associated biomass consumption (Fig. 5b). NOx emissions follow a similar pattern, with the highest emissions located in northern Pakistan. The values range from 0.003 to 0.016 gNOx/m² in fire-affected areas, indicating that nitrogen oxides are heavily released during wildfire events (Fig. 5c). This pattern is consistent with higher fuel loads and recurrent burning in northern forest ecosystems, where combustion of nitrogen-containing biomass and litter can increase NOx release during fire years. The CH₄ emissions show a distinct spatial pattern, with emissions values reaching up to 0.6–1.2 gCH₄/m². The highest concentrations are observed in central Pakistan, particularly where fires have consumed dense biomass, resulting in elevated methane emissions (Fig. 5d). Elevated CH₄ in these areas is plausibly linked to differences in combustion conditions and fuel characteristics, as methane emissions tend to increase under relatively oxygen-limited (smouldering) combustion and in fuels/moisture conditions that promote incomplete oxidation. The emissions of SO₂ and NO₂ are shown in Figure S4.

Across the five provincial regions of Pakistan, emissions of carbon and nitrogen compounds varied significantly. The highest total emissions were observed in the northern and central regions, which experienced the most FF events during key years (2009, 2016, 2021, and 2022). Higher fire-attributable emissions in these areas are expected because forest ecosystems contain greater fuel loads and carbon density, so fire events can generate large per-area emission pulses even where urbanisation is limited. The discharge of GHG, such as COx, NOx, and CH₄, poses a serious threat to forest ecosystems, potentially leading to further environmental degradation and loss of biodiversity. The high rates of carbon and nitrogen emissions from fire-affected regions highlight the critical need for enhanced FF management strategies. Mitigating these emissions is essential to reducing their impact on global carbon cycles and mitigating climate change. Furthermore, understanding the relationship between fire events and emissions can inform sustainable forest management practices and policy development aimed at preserving carbon sequestration potential in these ecosystems.

### Comparative analysis of random forest and XGBoost models for forest fire prediction

**Fig. 6:**
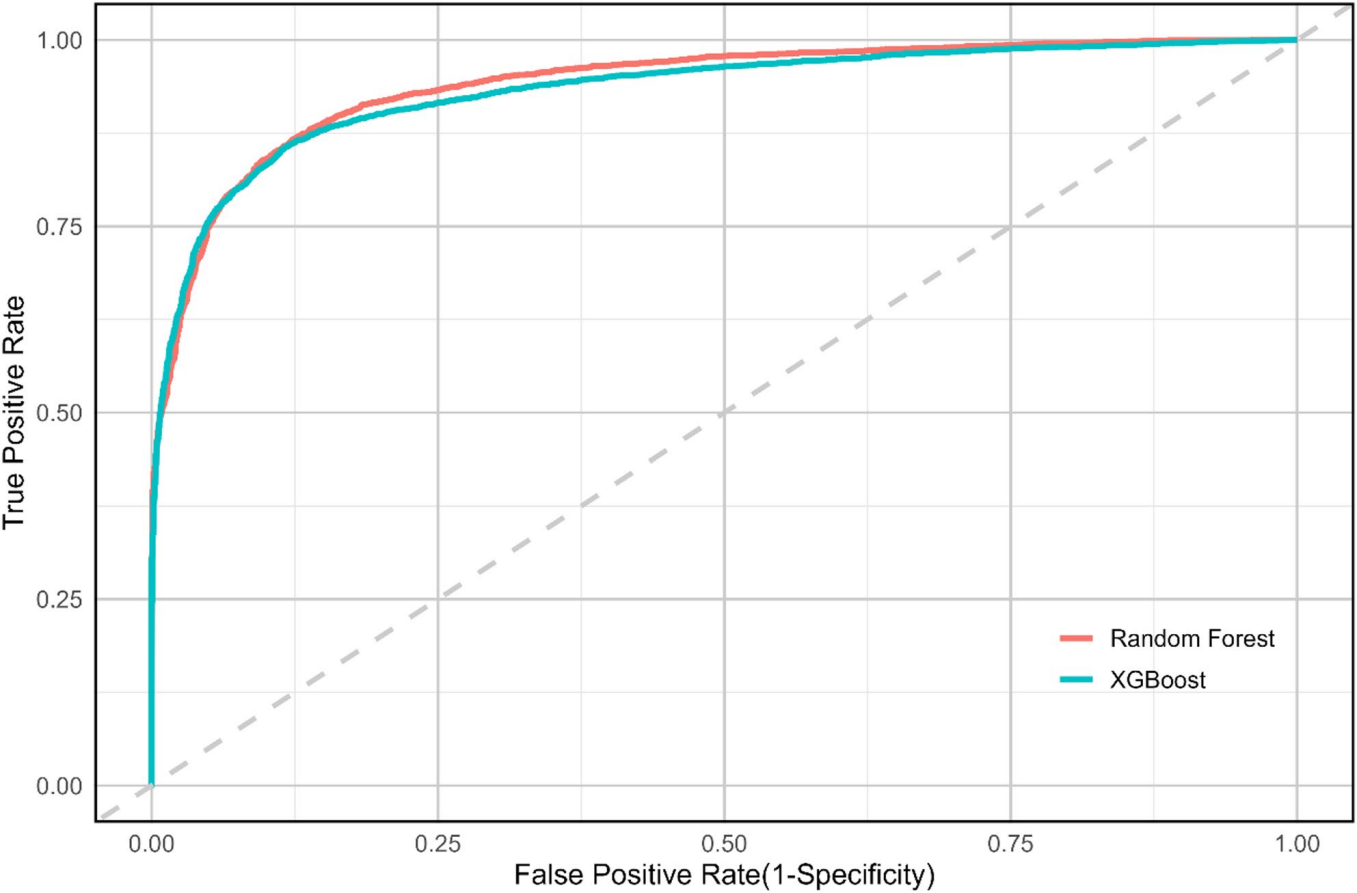
ROC (AUC) curves comparing the predictive performance of Random Forest and XGBoost

**Fig. 7:**
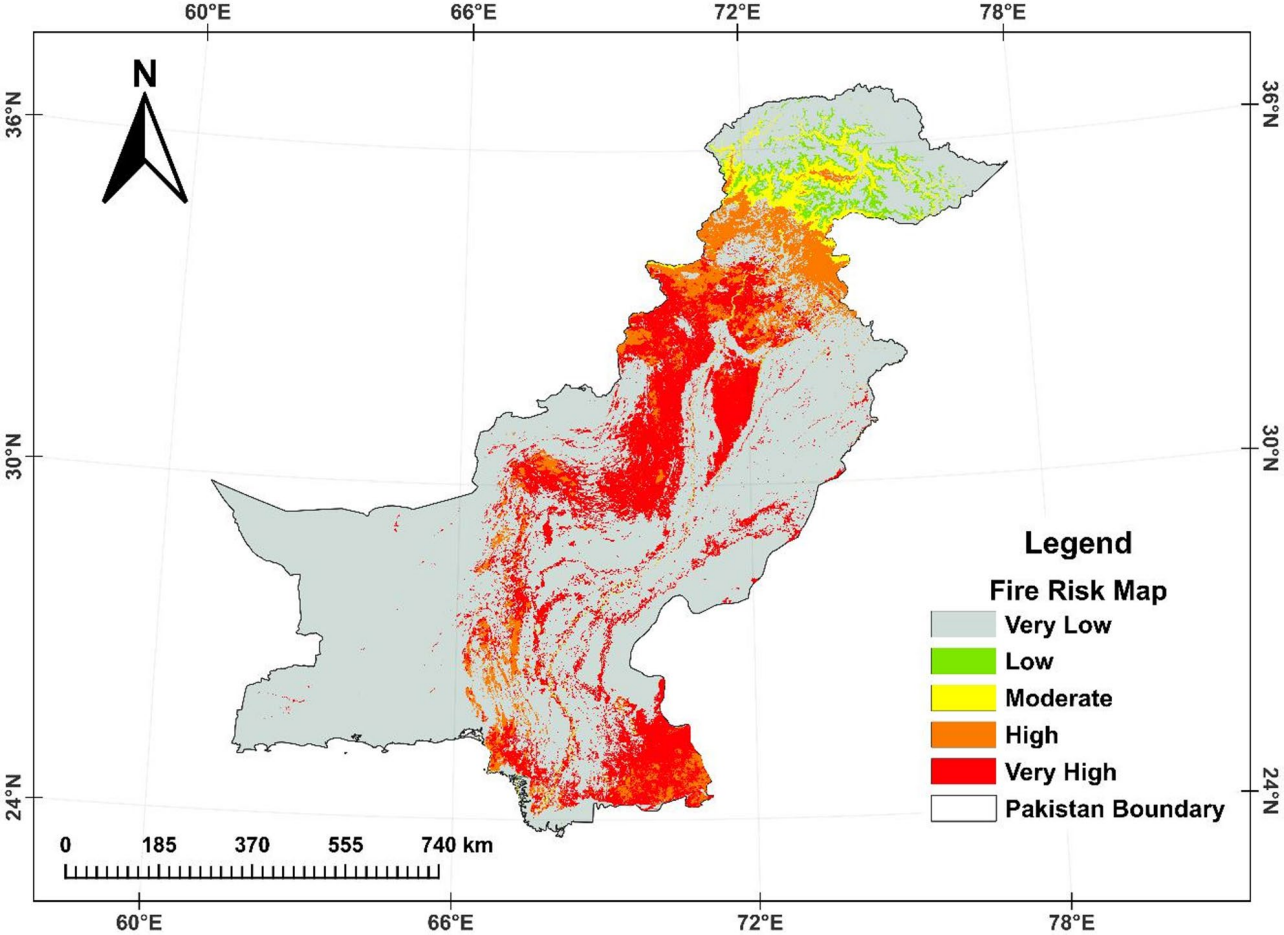
Wildfire susceptibility map of Pakistan (RF-based), classified into five susceptibility levels. Non-burnable land-cover classes (urban/built-up, water, permanent snow/ice, and barren land) were masked using prior to mapping

This study assessed multicollinearity among various environmental and topographic factors. The Tolerance (TOL) values for these factors were greater than 0.1, and the Variance Inflation Factors (VIF) were less than 10, indicating minimal covariance between the factors. This lack of multicollinearity suggests that these variables can be reliably used to assess fire risk within the study area’s defined parameters. The study employed an RF model to predict FF. After exploring the model’s configuration space, the highest accuracy was achieved, as detailed in Table 1. With optimized parameters, the RF model demonstrated an accuracy of 88.0% and an ROC–AUC of 0.938 (93.8%) (Table 1; Fig. 6). Here, accuracy represents the threshold-based classification correctness, whereas AUC summarises the model’s discriminatory ability across all thresholds. Figure 6 compares the RF model to the XGBoost model, revealing that the RF model outperformed XGBoost in terms of accuracy and AUC. Consequently, the RF model was identified as the most suitable for predicting FF in Pakistan (Fig. 7). Additionally, Figure S5 highlights the importance of various factors influencing forest fires, as assessed using SHAP values. The top five most influential variables are elevation, LAI, NDVI, wind speed, and LST, with other factors such as precipitation, NPP, soil temperature, population, and aspect also contributing to the model. Although the XGBoost model was also evaluated for accuracy and AUC to ensure its reliability across different conditions, it consistently ranked below the RF model. Table 1; Fig. 6 summarise the comparative performance of both models. This model, despite being less accurate than RF, can still contribute to improving fire management and mitigation strategies.

**Table 1:** Performance random forest and XGBoost

Model type	Accuracy (%)	AUC (0–1)	Precision (%)	Recall (%)	F1 Score (%)
Random forest	88.0	**0.938**	83.9	87.3	**85.6**
XGBoost	86.5	**0.929**	89.8	81.6	85.8

## Discussion

### Linkages between geographical variables, climate change, and forest fire events

The results of this study show clear associations between FFI and various geographical, climatic, and ecological factors across Pakistan, aligning with global trends observed in other regions such as India, Canada, and the United States. However, the drivers that shape these similarities differ across Pakistan’s physiographic zones because “fire” is jointly constrained by (i) fuel availability and continuity, (ii) fuel dryness and fire weather, and (iii) ignition pressure from human activity [1, 13, 45]. In northern Pakistan, the dense coniferous forests in KPK and GB experience higher fire intensities, especially in high-elevation areas where forests are more prone to wildfires. This is like findings in India, where high fire intensities are also observed in the deciduous and pine forests of the lower Himalayas [92]. In both regions, the inflammability of the dry forest litter, combined with extended dry seasons, contributes to frequent fires occurrences. A critical distinction is that the northern mountainous belt couples flammable fuels with topographic amplification of spread: steep slopes can promote upslope pre-heating and faster rates of spread, while complex valley–ridge systems can intensify local wind fields, making wind-driven runs more likely during dry pre-monsoon windows. Conifer- and pine-dominated stands (including resinous litter and needle fall) can maintain a persistent fine-fuel bed that dries rapidly, helping explain why high-elevation forest zones can show strong fire intensity despite cooler mean temperatures [47, 92].

As observed in other sub-tropical and temperate regions, climate change appears a key driver of increased fire intensity and frequency, with in Pakistan rising temperatures and decreased precipitation, particularly in central and southern regions, mirror patterns [89], where decreased moisture levels and extended dry spells contribute to larger and more frequent wildfires [26, 102]. This mechanism is most plausibly mediated through fuel aridity (vapour pressure deficit and soil–vegetation water stress), which increases the probability that a given ignition becomes a spreading fire; comparable evidence from other regions shows that warming-induced increases in fuel aridity and longer fire-weather seasons systematically elevate fire potential [1, 45]. These climate-driven changes exacerbate fire risks, especially in fire-prone areas such as Balochistan and Sindh, where dry scrubland and sparse vegetation provide ideal conditions for ignition and rapid-fire spread. Similar anthropogenic climate change has been identified as a major contributor to increasing FF events [11], Pakistan’s FF dynamics are likely influenced by both natural and human-induced factors. Importantly, sparse fuels can increase ignition probability but may limit severity where biomass is low; hence, “high-risk” areas may reflect frequent, fast-moving surface fires rather than high-severity crown fires. This distinction matters for interpreting both productivity losses and emissions, which scale strongly with fuel loads and combustion completeness. Land-use changes, such as deforestation and increased grazing, particularly in the central and northern regions, further intensifying the fire risks, as observed in studies of other fire-prone regions [80, 98]. Grazing and fuelwood collection can have opposing effects: they may reduce fine fuel loads locally, yet can also open canopy structure, dry the understorey, and expand human access corridors. Therefore, the observed spatial patterns are more consistent with a coupled “access–dryness–fuel continuity” mechanism than with a single-factor explanation. Recent Pakistan-focused evidence similarly indicates that human causes (negligence, land practices, and limited institutional capacity) combine with climatic constraints (notably precipitation variability) to shape hotspots in the northern highlands [71, 90]. The combination of topography, human interference, and climate factors reinforces the urgent need for targeted fire management strategies across Pakistan.

### Serviceability of satellite-based burn indices in ecosystem productivity and fire assessment

The spatial analysis of burn indices and their relationship to NPP highlights the practical value of satellite-based remote sensing tools for assessing fire impacts, as seen in [86]. In Pakistan, indices such as SAVI, LST, NMDI, LSWI, NBR, and MSAVI2 have proven effective in mapping fire-prone areas and measuring the ecosystem’s response to fire disturbances. This is consistent with research from regions such as the Iberian Peninsula and the boreal forests of Canada, where remote sensing tools like NDVI and SAVI have been employed to assess post-fire effects and burn severity [19, 81]. In northern Pakistan, the strong negative correlation between burn indices and NPP aligns with similar observations in regions like India and the Amazon, where wildfires have been shown to significantly reduce ecosystem productivity [29]. The high sensitivity of forests in GB and KPK to fire disturbances highlights the critical role of burn indices in identifying vulnerable zones and guiding fire prevention efforts. Mechanistically, indices incorporating surface energy and moisture constraints (e.g., LST- and moisture-sensitive metrics such as NMDI/LSWI) are expected to align more closely with fire susceptibility because they capture the immediate controls on fuel dryness and plant stress, whereas purely greenness-based indices can be confounded by phenology and post-monsoon regrowth. What sets this study from others is its use of the ΔNPP/ΔBurn approach, which allows for a deeper exploration of the spatial dynamics between burn intensity and productivity. Here, the ΔIndex/ΔNPP framework is interpreted as a sensitivity diagnostic rather than a causal coefficient: high values can arise either because burn impacts are strong or because baseline NPP is low and highly variable across elevation/land-cover gradients. Accordingly, we treat these maps as indicators of relative vulnerability and prioritisation, not as direct estimates of productivity loss per unit burn severity. While studies from other regions, such as the boreal forests of Canada, have demonstrated the long-term impacts of fires on carbon fluxes and forest recovery, the ΔIndex/ΔNPP method used here provides a more granular understanding of how small changes in fire behavior can lead to substantial shifts in productivity. This underscores the utility of remote sensing in FF management and reinforces the need for continuous monitoring of fire-prone areas [47]. We also acknowledge that dNBR-based severity signals can saturate in very high-severity burns and remain sensitive to pre-/post-fire compositing choices; therefore, uncertainty is larger in dense-canopy, high-biomass forests [86]. Consistent with the pooled statistics (Fig. S2), SAVI and MSAVI2 showed the strongest positive associations with NPP (*r* = 0.28 and 0.27), followed by LSWI and NMDI (*r* = 0.25), whereas NBR showed a weaker pooled association (*r* = 0.21). LST exhibited the strongest negative association with NPP (*r* = − 0.35), reflecting thermal stress constraints. The high inter-correlation among several indices (e.g., LSWI–NBR *r* ≈ 0.97) indicates partial redundancy; therefore, interpretation prioritises indices with clearer process linkage—NBR/LSWI for burn/moisture constraints and SAVI/MSAVI2 for soil-adjusted vegetation response—rather than relying on a single metric.

### Effects of FF on ecosystem Production, greenhouse gas Emissions, and carbon stocks

The findings on FF impact on ecosystem productivity and GHG emissions align with global research highlighting the destructive effects of fires on carbon sequestration and ecosystem services. This study shows that FF in Pakistan significantly reduces NPP, particularly in high-biomass areas like the northern regions, similar studies conducted in the boreal forests of Canada and tropical forests of Brazil [8, 100]. The stronger NPP reductions in northern forests are consistent with higher pre-fire biomass and canopy fuel continuity; where forests are denser, combustion and crown/understorey damage can produce larger and longer-lasting reductions in photosynthetic capacity than in low-biomass scrub and grass systems. The CASA model results show that fires not only reduce carbon sequestration potential but also lead to substantial carbon emissions, with emission values as high as 989 gC/m² in the most affected areas. The impact of FF on GHG emissions in Pakistan follows patterns observed in other fire-prone regions. In northern Pakistan, CO₂, NOx, and CH₄ emissions were found to be highest in areas with dense forest cover, mirroring findings from studies in India, Southeast Asia, and the Amazon basin [86]. This pattern is expected because emissions scale with (i) fuel load, (ii) burn severity/combustion completeness, and (iii) fire duration, while trace-gas partitioning varies with flaming versus smouldering phases; global syntheses such as GFED emphasise these constraints and provide a context for interpreting regional emissions estimates [99]. These emissions contribute to both local environmental degradation and global climate change, highlighting the need for enhanced fire suppression and reforestation efforts to mitigate these impacts. The comparison with global studies also underscores the importance of accounting for post-fire mortality and the long-term effects of fires on ecosystem recovery. In boreal and tropical forests, it can take decades for ecosystems to fully recover from large-scale fire events, and similar dynamics are likely at play in Pakistan’s northern forested regions.

### Comparative analysis of random forest and XGBoost models in fire prediction

The application of ML models, particularly RF and XGBoost, provides a strong approach to predicting FF risks in Pakistan. The superior performance of the RF model in this study, with an accuracy of 88.0% and an AUC of 93.4%, is consistent with findings from other studies that have employed RF for fire risk prediction (Table 1). Similar success was achieved in fire-prone regions of the Mediterranean and in Pakistan, where RF models outperformed other algorithms in predicting fire occurrence based on environmental variables [29, 89]. A likely reason is that RF performs well under predictor heterogeneity and non-linear interactions while remaining comparatively stable to noise and moderate multicollinearity conditions expected when combining topography, climate surfaces, and vegetation indices over complex terrain [12]. While XGBoost model has been effectively used in other environmental modeling applications, the slightly lower performance of XGBoost in this study could be due to the specific topographical and climatic conditions in Pakistan, which may be better captured by the RF model’s decision trees. This result highlights the importance of choosing appropriate ML techniques tailored to the specific characteristics of the study area. Interpreting SHAP results further supports a mechanistic reading of the model: elevation and aspect proxy fuel regimes and microclimate; LAI/NDVI represent live fuel amount and condition; wind speed captures spread potential; and LST reflects surface dryness and thermal stress together mapping onto the climate–fuel–people framework widely used to interpret fire regimes [13].

The RF-based susceptibility patterns (Fig. 7) are most plausibly explained by the combined effects of fuel availability, fire-weather, terrain controls, and human ignition pressure. The SHAP results support this mechanistic interpretation: elevation and aspect proxy microclimate and fuel regimes, LAI/NDVI represent live fuel amount and condition, wind speed captures spread potential, and LST reflects surface dryness and thermal stress. High-susceptibility zones in parts of KPK, AJK, GB, central Punjab, Sindh, and Balochistan therefore reflect the spatial co-occurrence of these drivers rather than forest cover alone, reinforcing the need for region-specific prevention strategies and targeted monitoring in areas where ignition likelihood and spread conditions coincide.

### Limitations and future directions

This study’s limitations include the use of satellite-derived indices, which may not fully capture fine-scale variations in fire behavior and ecosystem responses, potentially overlooking localized dynamics. Annual compositing can merge temporally separate burns into contiguous scars, and dNBR sensitivity can vary with phenology and sensor availability; therefore, sub-annual fire dynamics and repeated burns within a season may be under-resolved. Additionally, climatic variations such as rainfall over the period 2001–2023 were not extensively analyzed, limiting the understanding of their influence on fire intensity and NPP. While Random Forest and XGBoost models were employed, exploring other advanced machine learning approaches could enhance predictive capabilities, and the focus on select burn indices may miss other factors critical to understanding fire impacts on various vegetation types. Furthermore, the regional generalization of fire risk does not account for microclimatic or localized vegetation patterns, and the influence of controlled burns and agricultural practices on productivity was not quantified. Future research should incorporate high-resolution satellite data, detailed climatic variables, and a broader set of burn indices to improve predictive accuracy and understanding of fire–ecosystem dynamics. Employing deep learning techniques, such as CNNs or RNNs, could capture complex temporal patterns in fire behavior, and tailored regional fire management strategies are recommended to address localized fire risks effectively. Finally, incorporating explicit human-access predictors (distance to roads/settlements) and policy/management constraints could strengthen causal interpretation and improve operational relevance of risk mapping.

## Conclusion

This study quantified fire activity, productivity change, and fire-attributable emissions across Pakistan (2001–2023) and demonstrates pronounced spatial contrasts between the northern mountain forests and the arid–semi-arid lowlands. It shows that FF, particularly in the northern regions of KPK and GB, pose substantial risks to dense forest ecosystems, where high fuel continuity and complex terrain coincide with strong productivity losses and emission pulses during fire years. Among the evaluated remote-sensing indices, SAVI and MSAVI2 exhibited the strongest pooled associations with NPP (*r* = 0.28 and *r* = 0.27; *p* < 0.05), followed by LSWI and NMDI (*r* = 0.25), indicating that soil-adjusted greenness and canopy/soil moisture constraints provide the most reliable signals of productivity variability at the national scale (Fig. S2). LST showed the strongest negative association with NPP (*r* = − 0.35), highlighting the role of thermal stress and fuel dryness in constraining productivity. Although NBR showed a weaker pooled correlation (*r* = 0.21), its spatial patterns were diagnostically valuable in forested regions because it is directly sensitive to burn disturbance and post-fire canopy change, complementing moisture- and greenness-based metrics. The ΔNPP/ΔIndex framework further enabled spatially explicit interpretation of fire-related productivity sensitivity, indicating that productivity losses are most pronounced where burn/moisture stress signals (NBR/LSWI/LST) co-occur in northern forests, while southern shrubland/rangeland systems show more heterogeneous responses due to sparse fuels and seasonal phenology. Furthermore, the comparison of ML models indicates that RF outperforms XGBoost in predicting FF risks (accuracy = 88.0%, AUC = 0.938 vs. 86.5%, AUC = 0.929), supporting RF as a robust tool for national-scale fire susceptibility assessment in Pakistan. SHAP-based interpretation indicates that elevation, LAI/NDVI, wind speed, and LST jointly control predicted susceptibility, consistent with a coupled fuel–weather–terrain–human framework. Overall, the study provides an integrated, reproducible remote-sensing and machine-learning basis for prioritising fire monitoring and mitigation, particularly in high-biomass northern forests where fire-driven carbon losses and GHG emissions are most consequential. Future research should focus on refining predictive models using higher temporal resolution fire-weather data and assessing longer-term post-fire recovery trajectories to support sustainable fire management and climate mitigation in Pakistan’s diverse landscapes.

## Supplementary Information

Supplementary Material 1

## Data Availability

Data is provided within the manuscript or supplementary information files.
