# Supplementary material for: Remote sensing analysis of forest fire impacts on ecosystem productivity, greenhouse gas emissions, and fire risk in Pakistan
Source: Carbon Balance Manag. 2026 Feb 6;21:54. doi: 10.1186/s13021-026-00410-y (PMC12973728; doi:10.1186/s13021-026-00410-y)
Supplement: Supplementary file 1 — Supplementary Material 1 [file 13021_2026_410_MOESM1_ESM.docx]

Table S1: Explanations of independent Factors

| Category | Independent variables | Source | Rescale Resolution | Duration |
| --- | --- | --- | --- | --- |
| vegetation | Normalized Difference Vegetation Index | Landsat (5 TM, 7 ETM +, 8 OLI) | 30 m | 2001-2023 |
|  | Enhanced Vegetation Index |  |  |  |
|  | Leaf area index |  |  |  |
|  | Fraction of Photosynthetically Active Radiation |  |  |  |
| Climatic | minimum temperature | IDAHO_EPSCOR/TERRACLIMATE | 30 m | 2001-2023 |
|  | maximum temperature |  |  |  |
|  | wind speed |  |  |  |
|  | vapor pressure |  |  |  |
|  | Total precipitation |  |  |  |
|  | soil temperature |  |  |  |
|  | Land surface temperature | Landsat (5 TM, 7 ETM +, 8 OLI) | 30m | 2001-2023 |
| anthropogenic | Population density | https://hub.worldpop.org/ | 30m | 2001-2020 |
| Topographical | aspect | [https://earthexplorer.usgs.gov](https://earthexplorer.usgs.gov/) | 30m | 2020 |
|  | elevation |  |  |  |
| Fire indices | SAVI, LST, NMDI, LSWI, NBR, and MSAVI2 | Landsat (5 TM, 7 ETM +, 8 OLI) | 30m | 2001-2023 |

**Table S2: Forest Fire Intensity Levels and Area Coverage in Fire and Non-Fire Years.**

| Forest fire Intensity | Risk Level | Fire Years Area in km2 | Fire years Area in Percentage | Non-Fire Years Area in km2 | Non-Fire Years Area in Percentage |
| --- | --- | --- | --- | --- | --- |
| Very Low | 1 | 14990.90 | 1.81 | 85701.68 | 10.33 |
| Low | 2 | 642294.54 | 77.5 | 554340.11 | 66.81 |
| Moderate | 3 | 139724.57 | 16.86 | 109431.72 | 13.19 |
| High | 4 | 28189.12 | 3.4 | 78088.86 | 9.41 |
| Very High | 5 | 3583.42 | 0.43 | 2130.67 | 0.26 |


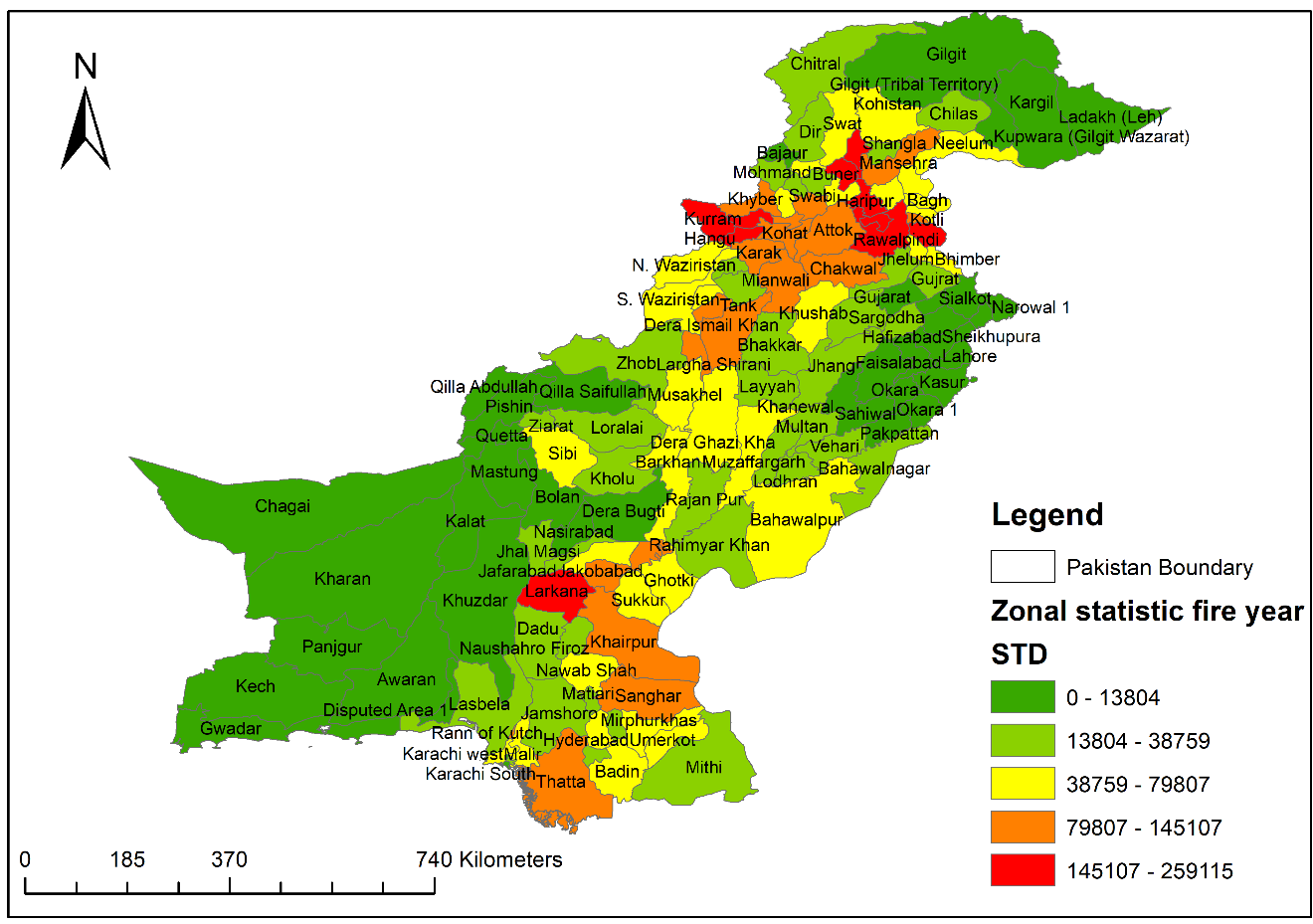


**Fig S1: variation in fire intensity is depicted in the standard deviation.**


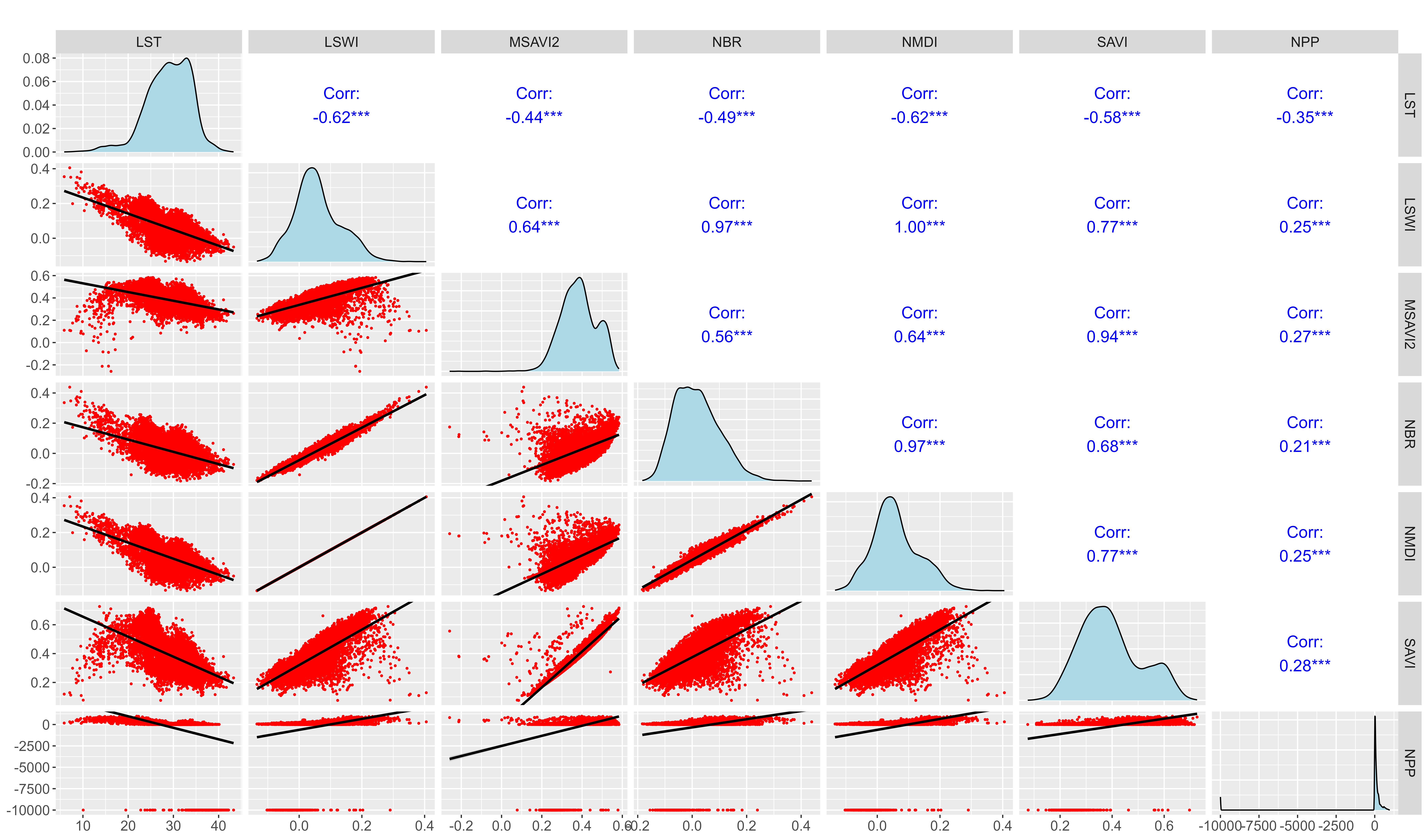


**Fig S2: Correlation matrix between burn indices and NPP. The statistical significance of the estimates was assessed as 0.05.**


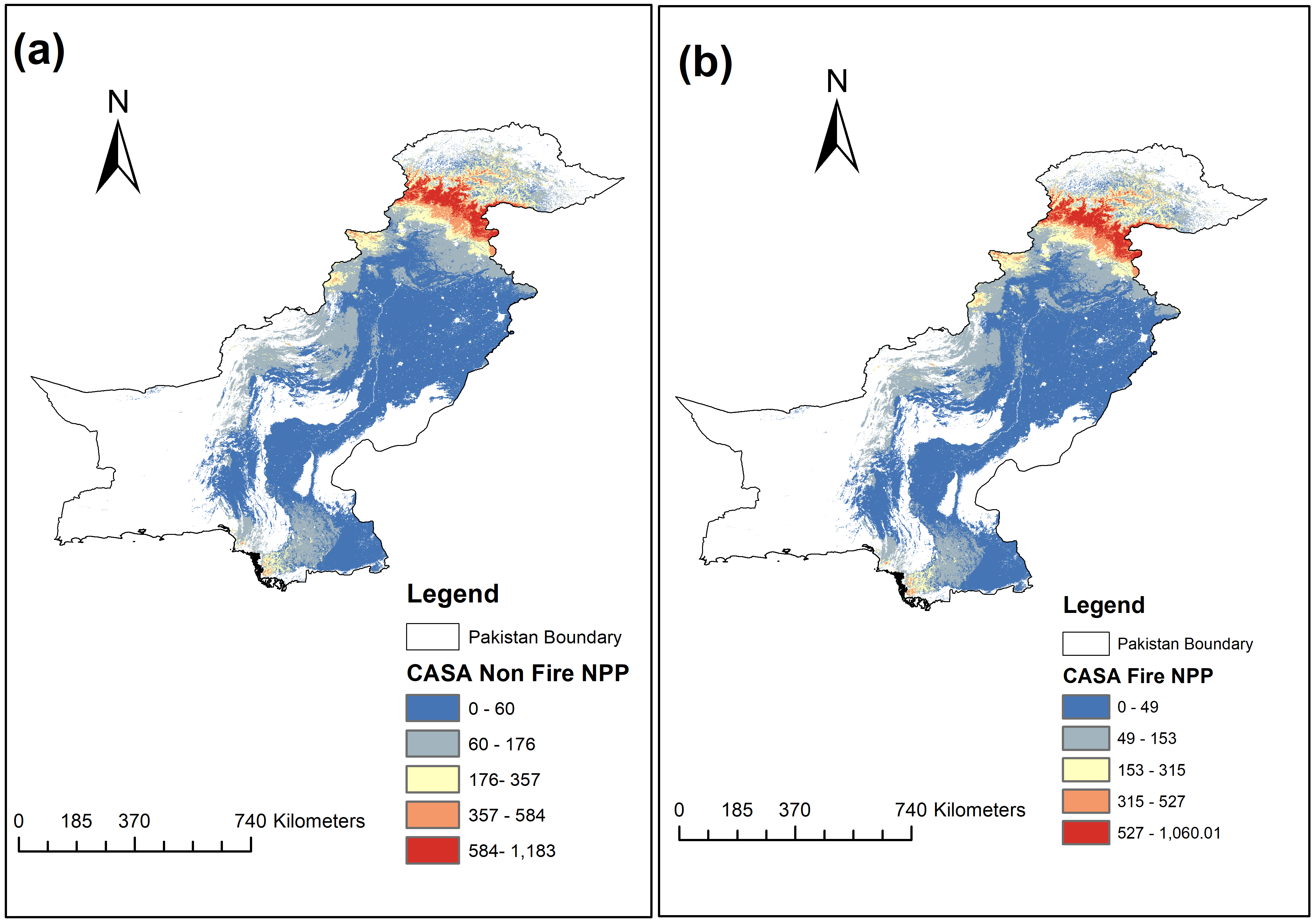


**Fig S3: CASA model NPP a) fire years b) non fire years**


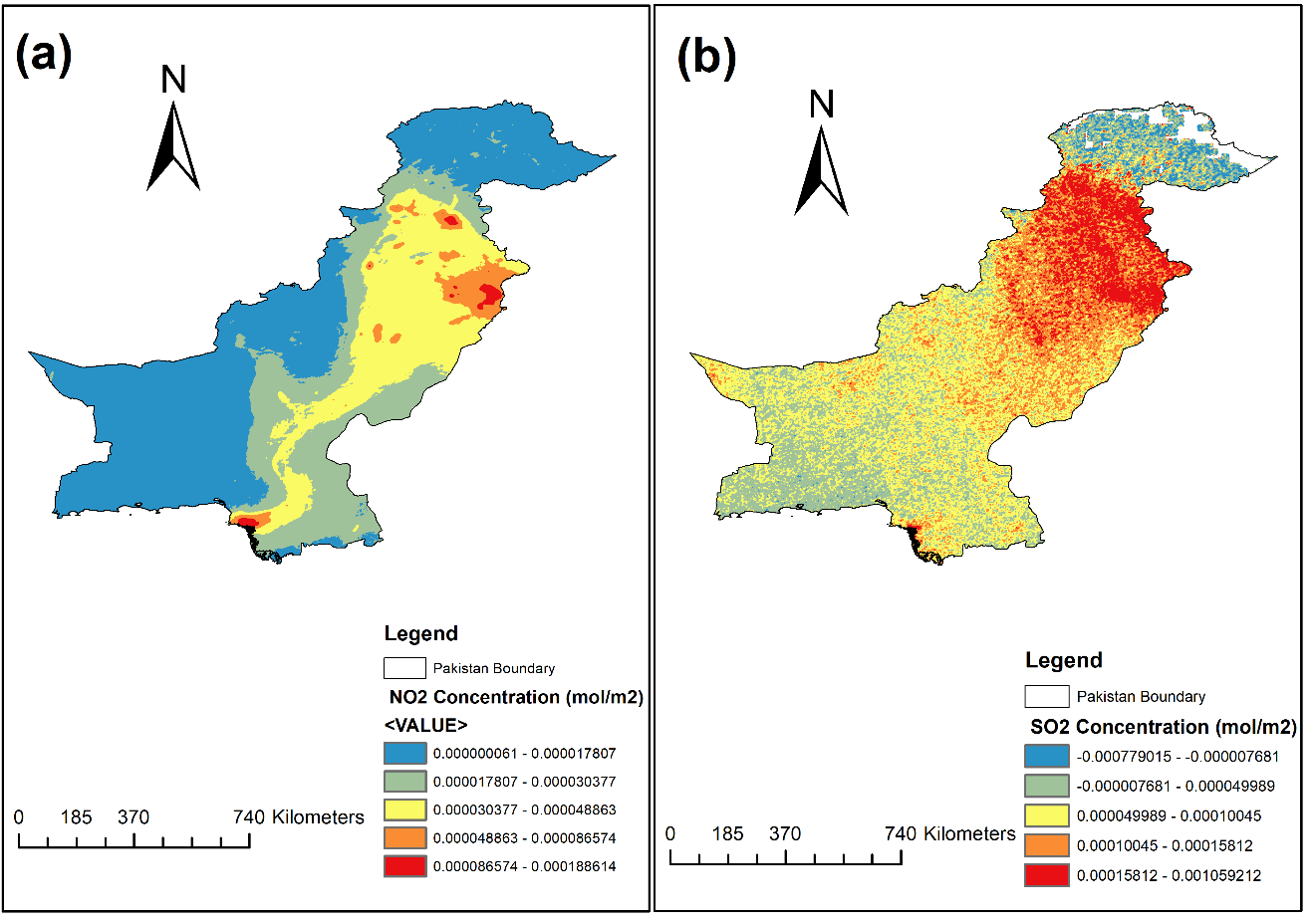


**Fig S4: Sentinel-5P NRTI concentrations of SO2 and NO2 in Pakistan**


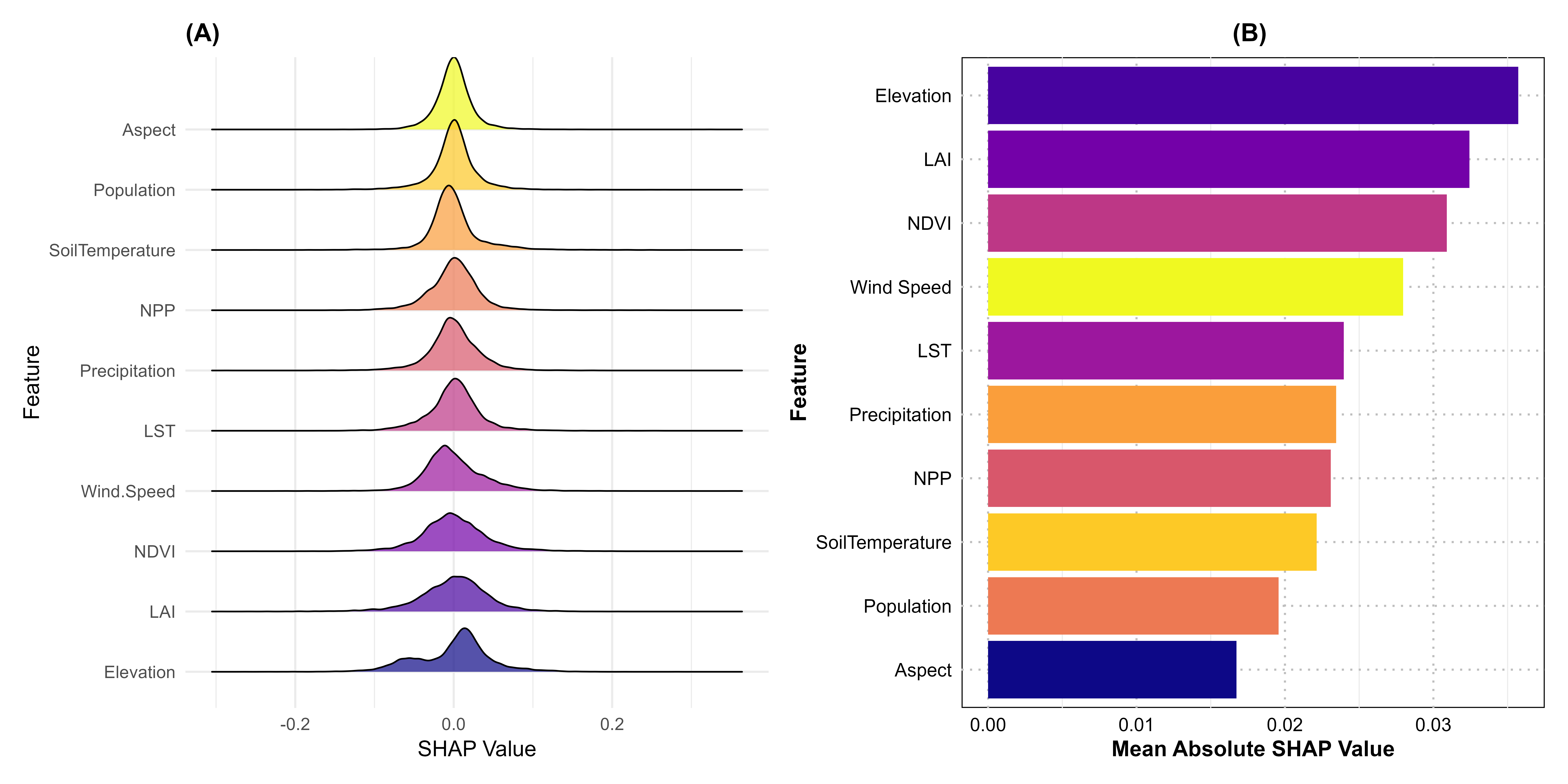


**Fig S5: variable importance influencing forest fire**

# Section 2

# Derivation of Vegetation Indices and LST

Vegetation indices and LST were derived from Landsat surface reflectance and thermal bands using the GEE platform for both fire and non-fire years. The selected vegetation indices included the NBR, SAVI, LSWI, NMDI, and MSAVI2, which capture vegetation stress, moisture variability, and post-fire impacts on ecosystem conditions (Avetisyan et al., 2023; Dindaroglu et al., 2021; Sannigrahi et al., 2020). The specific formulas used for calculating each index, along with their corresponding relationship with Net Primary Productivity (NPP) sensitivity analysis (ΔNPP/ΔIndex), are presented in Table 3.

**Table S3: Calculation of Burn Indices and their relationship with ΔNPP**

| Burn Index | Formula | ΔNPP Relationship |
| --- | --- | --- |
| NBR | (NIR - SWIR) / (NIR + SWIR) | ΔNPP / ΔNBR |
| SAVI | ((NIR - Red) / (NIR + Red + L)) × (1 + L) | ΔNPP / ΔSAVI |
| MSAVI2 | MSAVI2 = (2NIR+1-√((2NIR+1)^2^) - 8 (NIR-Red))/2 | ΔNPP / ΔMSAVI2 |
| NMDI | (NIR - (SWIR1 - SWIR2)) / (NIR + (SWIR1 + SWIR2)) | ΔNPP / ΔNMDI |
| LSWI | (NIR - SWIR) / (NIR + SWIR) | ΔNPP / ΔLSWI |
| LST | Derived from Landsat Thermal Bands | ΔNPP / ΔLST |

LST was estimated by Landsat thermal bands using the radiative transfer method within GEE, following the open-source approach developed (Ermida et al., 2020). This method incorporates atmospheric corrections, surface emissivity variations, and sensor-specific parameters to generate spatially explicit LST estimates at 30 m resolution. The algorithm has been widely validated for LST estimation from the Landsat series and is particularly suited for regional-scale fire impact assessment, vegetation stress analysis, and thermal anomaly detection.

# Validation of Fire Counts Derived from NBR using FIRMS Data

The accuracy of the Landsat-derived fire counts was assessed by validating the annual fire events mapped against active fire records provided by FIRMS. The FIRMS dataset, based on thermal anomaly detection, served as an independent reference to evaluate the reliability of NBR-based fire count (Aalto, 2020; Hu et al., 2014) estimation across Pakistan. The correlation analysis revealed a strong and statistically significant relationship between the NBR-derived fire counts and FIRMS fire points (r = 0.96, p < 0.001), indicating a high level of agreement (Figure 6). The error assessment metrics further supported the robustness of the methodology, with a Root Mean Square Error (RMSE) of 130.32 and a Mean Absolute Error (MAE) of 110.19. The bias between the two datasets was minimal (28.13), suggesting that the NBR-derived fire counts neither consistently overestimated nor underestimated the FIRMS observations. The scatter plot with the fitted regression line (Figure 6A) showed a consistent linear trend between the two datasets, while the year-wise comparison plot (Figure 6B) demonstrated similar temporal patterns in fire occurrences across the study period. The validation results confirmed the reliability of the Landsat-based NBR approach for annual fire count estimation, which is suitable for further analysis of fire intensity, vegetation response, and carbon emissions assessment.


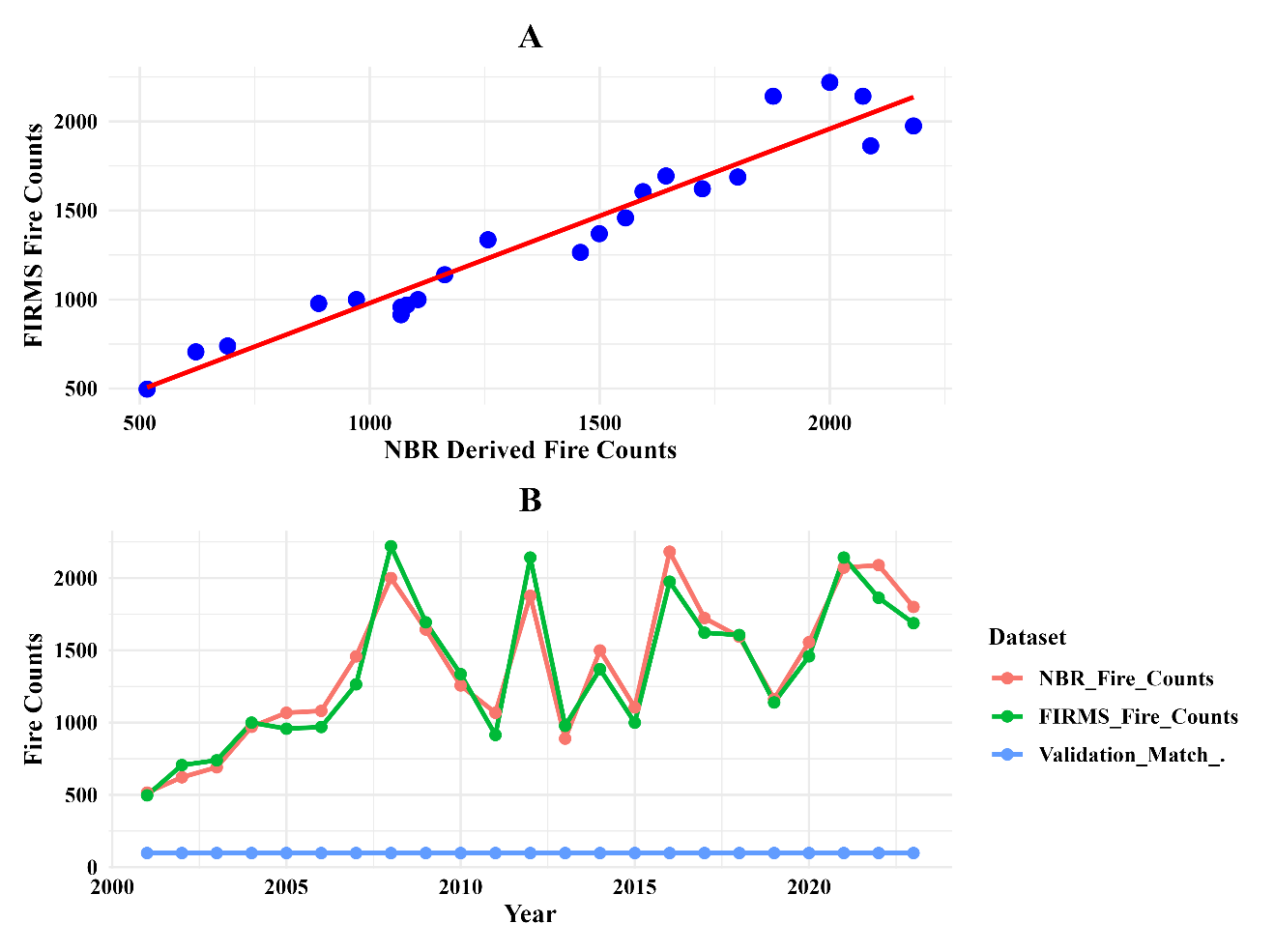


**Fig S6**: Validation of NBR-derived annual fire counts with FIRMS active fire records across Pakistan during 2000–2023. (A) Scatter plot illustrates the linear relationship between NBR-derived fire counts and FIRMS fire counts, showing a strong correlation (r = 0.96, p < 0.001). (B) Year-wise comparison of fire counts derived from NBR and FIRMS datasets, demonstrating consistent temporal patterns and trends in fire occurrences across the study period.

# Validation of CASA-Derived NPP Against MODIS NPP

The validation results of CASA-derived NPP were assessed separately for fire years and non-fire years by comparing extracted NPP values against MODIS NPP data. The statistical evaluation was conducted using the coefficient of determination (R²) and Root Mean Square Error (RMSE), which provide an indication of the model's predictive accuracy and error magnitude, respectively (Chai & Draxler, 2014; Mehmood, Anees, Muhammad, et al., 2024). For non-fire years, the CASA-derived NPP exhibited a strong positive correlation with MODIS NPP, yielding an R² = 0.7788 and an RMSE of 55.39 gC m⁻² year⁻¹ (Figure 7A). These results indicate a reasonable agreement between the two datasets in areas unaffected by fire disturbances, although some variability remains due to the inherent differences in model structure and spatial resolution. In contrast, for fire-affected years, the validation results demonstrated a slightly higher correspondence between CASA NPP and MODIS NPP, with an R² = 0.8191 and a lower RMSE of 50.08 gC m⁻² year⁻¹ (Figure 7B). The improved performance in five years is attributed to the strong response of vegetation productivity to fire-induced changes, which are well captured in both models. Overall, the validation results confirm that the CASA model provides a reliable estimation of spatial patterns and magnitudes of NPP across Pakistan for both fire and non-fire periods, with better predictive performance observed during fire years.


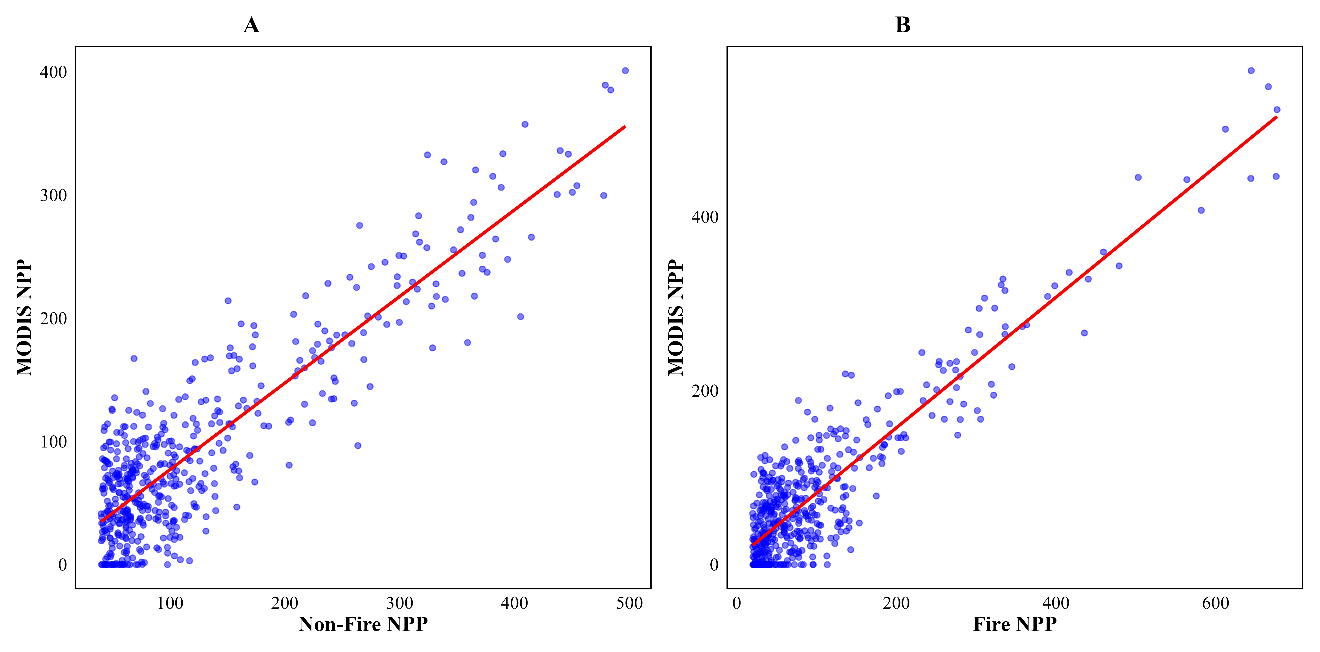


Fig. S7: Validation of CASA-derived Net Primary Productivity (NPP) against MODIS NPP for non-fire and fire years across Pakistan. (A) Scatter plot showing the relationship between non-fire year CASA NPP and MODIS NPP, with R² = 0.7788 and RMSE = 55.39 gC m⁻² year⁻¹. (B) Scatter plot showing the relationship between fire year CASA NPP and MODIS NPP, with R² = 0.8191 and RMSE = 50.08 gC m⁻² year⁻¹.The red line represents the fitted linear regression, while blue points denote the extracted NPP sample values. Both results indicate a strong agreement between CASA-derived NPP and MODIS NPP, with slightly improved model performance observed during fire-affected years.

Note:

**For CASA parameterisation, FPAR was computed from NDVI using Eq. (2) with** $a=1.164$ **and** $b=-0.143$ **(Myneni and Williams, 1994) and bounded to [0.001, 0.95].**
